# Vancomycin-resistant *Enterococcus* prevalence and its association along the food chain: a systematic review and meta-analysis

**DOI:** 10.1093/jac/dkaf008

**Published:** 2025-01-24

**Authors:** Benjamin Caddey, Waseem Shaukat, Karen L Tang, Herman W Barkema

**Affiliations:** Faculty of Veterinary Medicine, University of Calgary, Calgary, AB, Canada; Faculty of Veterinary Medicine, University of Calgary, Calgary, AB, Canada; Department of Medicine, Cumming School of Medicine, University of Calgary, Calgary, AB, Canada; Faculty of Veterinary Medicine, University of Calgary, Calgary, AB, Canada; One Health at UCalgary, University of Calgary, Calgary, AB, Canada

## Abstract

**Background:**

Vancomycin-resistant *Enterococcus* (VRE) are present across the One Health continuum and pose a considerable risk for transmission along the food chain. This systematic review and meta-analysis estimates the prevalence of VRE colonization in livestock, food of animal origin, and in human populations.

**Methods:**

Embase, MEDLINE and CAB Abstracts were searched for eligible literature. A total of 54 manuscripts passed inclusion criteria by providing prevalence estimates of VRE in a human population and at least one of either livestock or food. Random effects meta-analysis was conducted to determine prevalence estimates, and risk of bias in pooled estimates was assessed using funnel plots and Egger regression.

**Results:**

Global pooled prevalence of VRE colonization was highest in poultry and poultry meat at 16% (95% CI: 6%–28%) and 15% (95% CI: 1%–39%), respectively. Human-associated VRE colonization was highest in livestock workers, with a pooled prevalence of 11% (95% CI: 2%–25%), and lowest in the general public at 2% (95% CI: 0%–3%). Meta-regression demonstrated that human VRE prevalence increased at a rate of 0.75% (95% CI: 0.46%–1.04%; *P* < 0.001) per 1% increase in livestock VRE colonization.

**Conclusions:**

This meta-analysis established a clear link of VRE across One Health sectors. VRE colonization is likely elevated for those in contact with colonized animals or contaminated food products. Quality of evidence in pooled prevalence estimates was limited by publication bias and heterogeneity. The results of this study enhance calls for a One Health approach for mitigating the global burden of priority antimicrobial resistance pathogens.

## Introduction

Antimicrobial resistance (AMR) and resulting treatment-resistant infections were responsible for approximately 1.27 million human deaths worldwide in 2019, and these are projected to increase to 10 million annual deaths by the year 2050.^1^ Therefore, there is increasing urgency to understand sources of AMR and subsequent development of mitigation strategies through a One Health lens. Pressure for more prudent use of antimicrobials to curb selective pressure towards AMR development has been placed upon production animal medicine to alleviate the burden in human health.^2,3^ However, there are major gaps in knowledge of the clinical impact of priority antimicrobial-resistant pathogens in livestock on humans.

Individuals with direct exposure to livestock have increased risk of carrying antimicrobial-resistant bacteria.^4–6^ However, difficulties arise in exploring zoonotic transmission of AMR, because it requires an integrated surveillance approach that surveys all possible transmission pathways and intermediate sources/reservoirs for all pathogens of interest.^7^  *Enterococcus* spp. are commonly surveyed antimicrobial indicator organisms due to their ability to persist across all facets of the One Health landscape.^8^  *Enterococcus* spp. contain both commensal and pathogenic strains, and as inhabitants of the gastrointestinal tract, are privy to antimicrobial pressure and dissemination of AMR genes.^9^ As a high-priority AMR pathogen according to the WHO, vancomycin-resistant *Enterococcus* (VRE) is a deadly and highly transmissible pathogen.^10^ VRE isolates have been identified across One Health fields; however, strains associated with animals are often phylogenetically clustered with commensal *Enterococcus* strains, meaning that their risk of causing clinical infection is low.^8,11^ However, there is a large potential for VRE from animal sources to survive in humans and spread their AMR determinants to more pathogenic strains via horizontal gene transfer.^12–14^

VRE prevalence in the livestock industry is associated with avoparcin, a glycopeptide antibiotic used as a growth promoter,^15^ and personnel on exposed farms have a higher prevalence of VRE colonization.^16,17^ Even after the ban of avoparcin across Europe in the late 1990s, VRE are still isolated from various food and animal sources, highlighting the ongoing threat of potential VRE dissemination across One Health fields to date.^18,19^ As VRE can colonize throughout the food chain, it is important to survey VRE prevalence and potential dissemination mechanisms between animal sources and humans to develop mitigation strategies. Therefore, this systematic review and meta-analysis aimed to estimate the prevalence of vancomycin resistance in *Enterococcus* spp. isolated from livestock, food from animal origin, and humans globally. This review also aimed to characterize the association of VRE prevalence throughout the food chain, and explore factors related to increased prevalence rates.

## Materials and methods

### Search strategy

This systematic review was conducted in accordance with Preferred Reporting Items for Systematic Reviews and Meta-Analyses (PRISMA) guidelines.^20^ Three identified databases—MEDLINE (Ovid), CAB Abstracts (EBSCO) and Embase (Ovid)—were systematically searched in October 2022 and then again in May 2024 using a thoroughly defined search strategy (Table S1, available as Supplementary data at *JAC* Online) with no language or date restrictions. The search strategy was built with the assistance of an academic librarian. References of included literature, including surveillance reports and grey literature, were also searched and considered for inclusion. Search strings were created with the PICO (Population, Intervention, Comparison, Outcome) framework in mind using terms encompassing VRE, antimicrobial-resistant *Enterococcus*, and an extensive list of livestock and food terms targeting several animal species.

### Study selection

**Figure 1.**
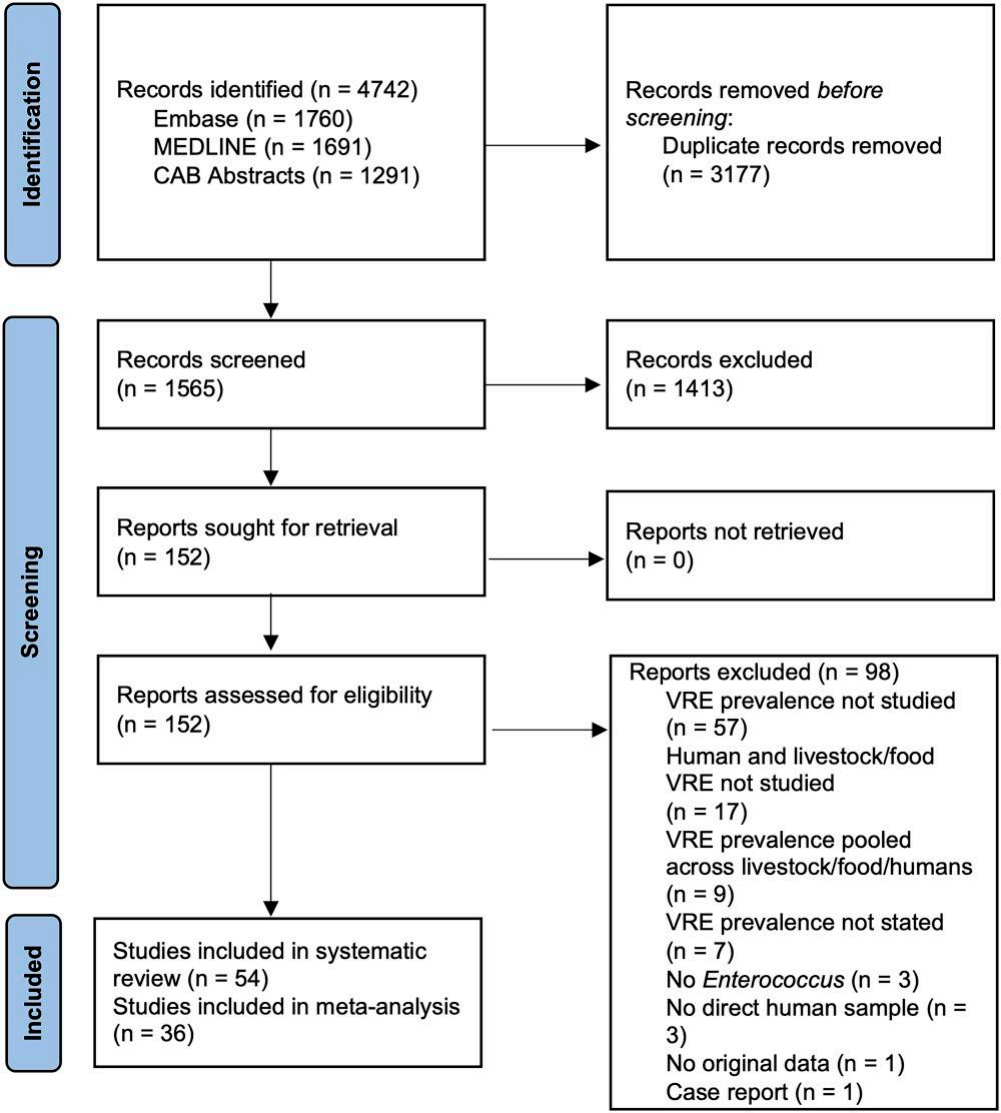
PRISMA flowchart.^20^ Number of studies identified, screened and included in systematic literature search of three databases. All literature that met inclusion criteria was included in the systematic review, and those studies that stated VRE colonization prevalence were included in the quantitative meta-analysis.

Two independent reviewers (B.C. and W.S.) conducted title and abstract screening of the records retrieved from the search strategy. Records were excluded if (i) they did not isolate *Enterococcus* spp., (ii) they did not investigate vancomycin resistance of *Enterococcus*, (iii) they did not determine VRE prevalence in humans, (iv) they did not determine VRE prevalence in either livestock or food, or (v) they did not include original data (Figure 1). Thereafter, full-text review was conducted separately by both B.C. and W.S. At this stage, studies were excluded using the same criteria and additionally if (i) they did not report prevalence estimates or other measures of association from which prevalence could be calculated, (ii) they reported pooled prevalence of humans and animals/food instead of separately reporting, or (iii) vancomycin susceptibility was not separately reported among other antibiotic susceptibilities (i.e. only MDR reported). Disagreements at both the title and abstract screening and full-text screening stages were resolved through mutual consensus by both reviewers.

### Data extraction and quality assessment

Both B.C. and W.S. independently extracted data from the studies that met inclusion criteria using Microsoft^®^ Excel. Data extracted included VRE prevalence for each *Enterococcus* species mentioned (total VRE over total *Enterococcus* tested), country of subject origin, specimen type, antimicrobial susceptibility testing (AST) method and MIC cut-off, and VRE genotyping information. A full outline of data extracted is included in Tables S2 and S3. Disagreements in data extraction were solved by mutual consensus. Quality assessment was performed using the Newcastle–Ottawa quality assessment scale adapted for cross-sectional studies.^21,22^ The studies were graded based on sample selection (representativeness, sample size) and assessment of outcome with a maximum score of 4. Representativeness of sample was assessed based on sampling strategy, with a full score for representative sampling of target population based on random or systematic sampling of individuals. Sample size for included literature was justified when a sample size calculation was stated. Outcome assessment quality received a full score if externally validated laboratory guidelines for AST were used.

### Data synthesis and analysis

All statistical analyses were performed using R version 4.2.1.^23^ All studies that passed screening were included in systematic review. However, for inclusion in quantitative meta-analysis, VRE were defined as *Enterococcus* spp. with a vancomycin MIC  ≥32 mg/L. This breakpoint captures human clinical resistance (as defined by the CLSI guidelines)^24^ and accounts for potential sources of heterogeneity by removing less clinically relevant isolates exhibiting low-level vancomycin resistance (particularly *vanC*-mediated resistance).^15^ Additionally, only studies estimating VRE prevalence at colonization level (rather than isolate- or herd-level resistance) were included in quantitative meta-analysis.

We pooled prevalence estimates for VRE colonization of humans, livestock (all species) and food (all animal species) separately. A random effects meta-analysis using the inverse variance method was performed using the ‘meta’ v. 6.0.0 package.^25^ Individual point estimates were transformed using the double arcsine method to adjust for extreme prevalence estimates and to ensure CIs fell within 0 and 1. Pooled VRE prevalences in each of humans, livestock and food were estimated along with 95% CIs, whereas 95% prediction intervals (PIs) were also calculated for better understanding of the heterogeneity of pooled prevalence estimates. The *I*^2^ statistic was used to estimate heterogeneity among included studies. In anticipation of high heterogeneity between studies, stratified analysis was performed on extracted study variables including for each subgroup based on originating sample population demographics and animal species. For humans, subgroups were general public, hospital-associated (clinical samples, healthcare workers, in/outpatients), livestock workers, and slaughterers (including butchers and retail staff). For livestock, subgroups were cattle, pigs, poultry and others (e.g. small ruminants: goats and sheep). Food products were split into dairy products, fish, poultry and red meat (pork and beef). Meta-regression was also used to estimate the association of VRE colonization prevalence with study characteristics including subgroup, publication year, continent, sample type and AST method.

Meta-regression was also used to estimate the association of human VRE prevalence with livestock and food VRE prevalence, using prevalence estimates from each study that contained estimates for both humans and animals/food. For this, only one prevalence estimate from either livestock or food was used for each study (if multiple estimates were reported in a study, then VRE counts were combined into a single prevalence estimate). All human population subgroups were still included to allow for potential differences in association for each human population. Finally, small study effects were explored using funnel plots and the Egger regression test.^26^ Confidence in pooled prevalence estimates was assessed using the GRADE (grading of recommendations assessment, development and evaluation) framework,^27^ where risk of bias, imprecision, inconsistency, publication bias and indirectness were considered for a final GRADE score.

## Results

### Study identification, characteristics and quality

A total of 1565 unique studies were identified from the systematic search of MEDLINE, Embase and CAB Abstracts (Figure 1). Most (*n* = 98) of 152 records included in the full-text review were ultimately excluded due to lack of VRE prevalence estimates or only reporting VRE estimates for one part of the food chain (Figure 1). For inclusion in systematic review, 54 studies were identified, and 36 of these studies reported VRE (MIC ≥ 32 mg/L) colonization prevalence and were thus included in the meta-analysis (Figure 1). There was a very good inter-rater agreement between the two reviewers (κ = 0.72) for the title and abstract screening, and a good inter-rater agreement (κ = 0.49) for the full-text review stage.

A reference list of included literature is available in the Supplemental material. The included literature mostly comprised journal manuscripts (Table 1). European studies accounted for 63% (*n* = 34/54) of the included studies (Table 1). Asia, Africa and North America each represented ≤15% of total included studies, with no representation from South America or Australia (Table 1). VRE prevalence in humans was one of the inclusion criteria; therefore, all included studies reported this estimate. The number of studies that reported VRE prevalence in food-production animals was more than double the number of studies that reported food VRE prevalence estimates (Table 1). The unit of analysis for prevalence estimates was most often at the animal/food/human colonization level (*n* = 42 studies), although several studies presented both isolate- and colonization-level prevalence estimates (Table 1). Previous glycopeptide exposure of humans/animals was infrequently reported in VRE prevalence estimates (*n* = 5; Table 1).

**Table 1.** Characteristics of the studies included for systematic review

	No. (%) studies
Article type	
Journal manuscript	47 (87)
Thesis	1 (2)
Research letter	5 (9)
Government report	1 (2)
Continent	
Africa	8 (15)
Asia	8 (15)
Europe	34 (63)
North America	4 (7)
Target population^a^	
Humans	54 (100)
General public	25 (46)
Hospital-associated	22 (41)
Slaughterhouse workers^b^	8 (15)
Livestock workers	15 (28)
Livestock	42 (78)
Swine	19 (35)
Poultry	28 (52)
Cattle	17 (31)
Other^c^	5 (9)
Food	20 (37)
Red meat	8 (15)
Poultry	12 (22)
Dairy	8 (15)
Fish	3 (6)
Sample type^a^	
Faeces	42 (78)
Clinical specimen	14 (26)
Skin	5 (9)
Milk	8 (15)
Meat	14 (26)
Not stated	1 (2)
Unit of analysis^a^	
Isolate	29 (54)
Livestock/person/food product	42 (78)
Herd/flock	3 (6)
Glycopeptide exposure^a^	
Yes	5 (9)
No	4 (7)
Not stated	52 (96)
Susceptibility testing method^a^	
Broth microdilution	13 (24)
Agar dilution	5 (9)
Disc diffusion	10 (19)
Selective VRE agar	29 (54)

^a^Each study can be included in more than one category.

^b^Includes slaughterhouse workers, butchers, retailers.

^c^Goat, sheep.

The 54 included studies were assessed using an adapted Newcastle–Ottawa scale on three main metrics. Sampling strategies for the majority of included literature (69%) generally led to truly or somewhat representative estimates of target populations (Table 2 and Table S4). Convenience sampling or lack of clarity in sampling strategy was still relatively common when VRE prevalence was estimated (Table 2). Most studies used the CLSI or EUCAST guidelines for AST (Table 2). Sample size justification for VRE prevalence estimation was reported for 1 of the 54 studies (Table 2).

**Table 2.** Quality assessment summary

	No. (%) studies
Representativeness of sample	
Truly or somewhat representative of target population	37 (69)
Convenience sampling or no description	17 (31)
Sample size	
Justified	1 (2)
Not discussed	53 (98)
Outcome assessment	
Objective, validated laboratory AST	39 (72)
Non-standard AST	14 (26)
No description of laboratory methods	1 (2)

### VRE species distribution

VRE as defined in each study based on individual MIC breakpoints (Tables S1 and S2) demonstrated clear species distribution trends across the food chain (Table 3). Across all human subgroups, VRE species were dominated by *E. faecium*, accounting for at least 50% of all VRE isolates identified (Table 3). This predominance of vancomycin-resistant *E. faecium* was also reflected in swine and poultry, while being less represented in red meat, poultry meat and cattle (Table 3). Vancomycin-resistant *E. faecalis* were most prevalent in hospital-associated samples, livestock workers and poultry meat (Table 3). Vancomycin resistance in *Enterococcus* spp. other than *E. faecium* and *E. faecalis* represented less than 20% of hospital-associated VRE colonization (Table 3). However, these *Enterococcus* spp. represented approximately 42% of VRE colonization in the general public, 31% of livestock workers, 45% in pigs and 35% in poultry animals, and reflected the majority of VRE species collected from food samples (Table 3).

**Table 3.** Distribution of vancomycin-resistant *Enterococcus* species counts recovered from each target population^a^

Target population	Species count, *n* (%)				
	*E. faecium*	*E. faecalis*	*E. hirae*	*E. gallinarum*	*Enterococcus* spp.
Humans					
General public	42 (50)	7 (8)	3 (4)	22 (26)	10 (12)
Hospital-associated	118 (56)	60 (28)	1 (1)	21 (10)	11 (5)
Slaughterhouse workers	18 (65)	2 (7)	4 (14)	4 (14)	0 (0)
Livestock workers	34 (55)	9 (14)	11 (17)	7 (11)	2 (3)
Livestock					
Swine	55 (45)	13 (10)	0	27 (22)	29 (23)
Poultry	332 (58)	41 (7)	12 (2)	140 (25)	47 (8)
Cattle	1 (2)	0	1 (2)	9 (18)	40 (78)
Other^b^	NT	NT	NT	NT	NT
Food					
Red meat	11 (28)	3 (8)	0	16 (41)	9 (23)
Poultry	9 (19)	14 (29)	0	22 (46)	3 (6)
Dairy	NT	NT	NT	NT	NT
Fish	NT	NT	NT	NT	NT

NT, no species typing available.

^a^VRE MIC breakpoints are defined at the study level and are located in Table S1.

^b^Goats, sheep.

Only 11 of the 54 studies performed genotyping of VRE strains beyond normal species typing (Table S3). *E. faecium* was the most commonly typed species. Two hospital-associated *E. faecium* isolates were typed and represented ST774, which were not reported in livestock or food VRE. Studies investigating PFGE profiles of VRE did not demonstrate related strains between hospital-associated and livestock/food isolates. Shared VRE STs and PFGE genotypes were most frequently observed between the general public/farmers and livestock/food. Vancomycin-resistant *E. faecium* ST8 and ST195 were isolated from poultry, poultry meat, livestock workers and the general public. Vancomycin-resistant *E. faecalis* ST116 was isolated from both poultry meat and the general public (Table S3).

### Pooled prevalence of VRE in humans, livestock and food

Forest plots of pooled prevalence estimates for VRE colonization in each of humans, livestock and food are displayed in Figures S1–S3. The 45-point prevalence estimates for humans resulted in a pooled VRE colonization prevalence of 5% (95% CI: 2%–8%), with an *I*^2^ of 95% (Table 4). VRE colonization prevalence was highest in livestock workers (11%; 95% CI: 2%–25%; *I*^2^: 95%) followed by hospital-associated (8%; 95% CI: 2%–18%; *I*^2^: 95%); meanwhile, it was lowest in the general public (2%; 95% CI: 0%–3%; *I*^2^: 92%), which also had the narrowest 95% PI of between 0% and 13% (Table 4 and Figure S1).

**Table 4.** Subgroup and pooled prevalence estimates of vancomycin-resistant (MIC ≥ 32 mg/L) *Enterococcus* colonization

Target population	No. point estimates^a^	Pooled proportion (95% CI)	*I* ^2^ | τ^2^
Humans	45	0.05 (0.02–0.08)	95% | 0.0378
General public	18	0.02 (0.00–0.03)	92% | 0.0110
Hospital-associated	13	0.08 (0.02–0.18)	95% | 0.0653
Livestock workers	7	0.11 (0.02–0.25)	95% | 0.0585
Slaughterhouse workers	7	0.05 (0.00–0.16)	90% | 0.0424
Livestock	39	0.06 (0.02–0.11)	97% | 0.0657
Cattle	9	0.02 (0.00–0.11)	87% | 0.0650
Swine	12	0.03 (0.00–0.07)	90% | 0.0274
Poultry	15	0.16 (0.06–0.28)	98% | 0.0796
Other^b^	3	0.00 (0.00–0.01)	0% | 0
Food	17	0.07 (0.01–0.17)	96% | 0.0756
Dairy	2	0.02 (00–0.12)	87% | 0.0195
Fish	2	0.05 (0.00–0.42)	91% | 0.0975
Poultry	7	0.15 (0.01–0.39)	98% | 0.1173
Red meat	6	0.04 (0.00–0.15)	90% | 0.0469

τ^2^ statistic reflects the variance in VRE prevalence estimates between studies.

^a^Some studies measure multiple subgroups, thus are included multiple times in each target population.

^b^Goats, sheep.

Pooled prevalence for livestock VRE colonization was slightly higher than for humans, at 6% (95% CI: 2%–11%; *I*^2^: 97%). VRE prevalence was lowest in cattle (2%; 95% CI: 0%–11%; *I*^2^: 87%) and swine (3%; 95% CI: 0%–7%; *I*^2^: 90%), and highest in poultry (16%; 95% CI: 6%–28%; *I*^2^: 98%) (Table 4). Forest plot analysis demonstrated a decrease in poultry VRE colonization proportion from 1997 to 2019 (Figure S2).

A total of 17 point prevalence estimates were obtained from 14 studies reporting on food VRE colonization and were used in pooled prevalence estimation (Figure S3). The pooled VRE colonization estimate for food was 7% (95% CI: 1%–17%; *I*^2^: 96%) (Table 4). VRE colonization was highest (15%: 95% CI: 1%–39%; *I*^2^: 98%) in poultry meat (Table 4). Poultry meat was the largest source of heterogeneity in pooled VRE prevalence estimates of the food chain, with a 95% PI spanning 0%–97% (Figure S3). Red meat, which included pork and beef, had a pooled VRE colonization prevalence similar to other livestock at 4% (95% CI: 0%–15%; *I*^2^: 90%), whereas dairy and fish isolates only had two studies reporting VRE prevalence estimates (Table 4).

Substantial risk of publication bias was evident for each pooled prevalence estimate (Figures S4–S6). Included studies often skewed towards extreme prevalence estimates when SEs of the estimates were low, based on visual assessment of funnel plots. Human and food pooled prevalence estimates had evidence of publication bias (Egger regression tests: *P* < 0.0001 and *P* = 0.04, respectively), whereas there was no statistically significant evidence for publication bias for pooled livestock estimates (*P* = 0.39).

### Impact of study characteristics on VRE prevalence estimates

Meta-regression was used to evaluate the impact of study variables on VRE colonization prevalence estimates. Compared with the general public, VRE colonization was higher for hospital-associated samples (*P* = 0.03), livestock workers (*P* = 0.006) and slaughterers/retailers (*P* = 0.007) (Table 5). VRE colonization prevalence in humans appeared to decrease by publication year when inspecting forest plots for livestock workers and slaughterers (Figure S1), but this was not significant (*P* = 0.18) in the meta-regression for all human samples (Table 5). VRE colonization in humans was highest in Africa (Table 5), but this difference was not statistically significant when compared with other continents. Sample type and AST method both impacted VRE colonization prevalence estimates (Table 5). Prevalence was higher in faecal samples than skin samples (*P* = 0.049); meanwhile, studies employing disc diffusion techniques had a higher VRE colonization prevalence compared with studies using agar dilution (*P* = 0.04; Table 5).

**Table 5.** Meta-regression of study variables on vancomycin-resistant (MIC ≥ 32 mg/L) *Enterococcus* colonization

Variables		Estimate (95% CI)	*P* value
**Humans**			
Subgroup	General public	Ref.	
	Hospital-associated	0.179 (0.014 to 0.344)	0.033
	Livestock workers	0.224 (0.064 to 0.384)	0.006
	Slaughterers and retailers	0.287 (0.079 to 0.495)	0.007
Year		−0.007 (−0.018 to 0.003)	0.179
Continent	Africa	Ref.	
	Asia	−0.213 (−0.503 to 0.076)	0.150
	Europe	−0.141 (−0.435 to 0.153)	0.348
	North America	−0.258 (−0.617 to 0.100)	0.158
Sample type	Skin	Ref.	
	Clinical	0.347 (−0.007 to 0.702)	0.055
	Faecal	0.339 (0.000 to 0.678)	0.049
	Milk	0.570 (−0.138 to 1.278)	0.114
AST method	Agar dilution	Ref.	
	Disc diffusion	0.371 (0.012 to 0.730)	0.043
	Broth microdilution	0.082 (−0.275 to 0.440)	0.652
	Selective VRE agar	0.155 (−0.169 to 0.480)	0.348
**Livestock**			
Subgroup	Cattle	Ref.	
	Swine	0.127 (−0.090 to 0.345)	0.250
	Poultry	0.352 (0.149 to 0.556)	0.001
	Other^a^	−0.069 (−0.414 to 0.276)	0.694
Year		−0.014 (−0.032 to 0.004)	0.120
Continent	Africa	Ref.	
	Asia	−0.238 (−0.561 to 0.086)	0.150
	Europe	−0.222 (−0.560 to 0.116)	0.198
	North America	−0.109 (−0.475 to 0.256)	0.557
Sample type	Milk	Ref.	
	Faecal	−0.385 (−0.803 to 0.034)	0.072
	Other^b^	−0.726 (−1.200 to −0.252)	0.003
AST method	Agar dilution	Ref.	
	Disc diffusion	0.415 (−0.048 to 0.878)	0.079
	Broth microdilution	0.126 (−0.327 to 0.580)	0.585
	Selective VRE agar	0.117 (−0.318 to 0.553)	0.598
**Food**			
Subgroup	Red meat	Ref.	
	Dairy	0.266 (−0.568 to 1.100)	0.532
	Fish	0.044 (−0.339 to 0.426)	0.823
	Poultry	0.021 (−0.239 to 0.280)	0.876
Year		−0.016 (−0.048 to 0.016)	0.334
Continent	Africa	Ref.	
	Asia	−0.707 (−1.345 to −0.069)	0.030
	Europe	−1.096 (−1.941 to −0.250)	0.011
	North America	−1.547 (−2.474 to −0.619)	0.001
Sample type	Dairy product	Ref.	
	Meat	0.360 (−0.272 to 0.993)	0.264
AST method	Agar dilution	Ref.	
	Disc diffusion	0.133 (−0.443 to 0.708)	0.651
	Broth microdilution	0.262 (−0.150 to 0.674)	0.212
	Selective VRE agar	0.494 (−0.023 to 1.010)	0.061

^a^Goats, sheep.

^b^Cloaca.

Poultry were most frequently colonized by VRE (Table 4 and Figure S2), and more often colonized by VRE (*P* = 0.001) than cattle (Table 5). Similar to humans, poultry VRE colonization prevalence appeared to decrease by publication year (Figure S2), but this association was not statistically significant (*P* = 0.12; Table 5). Laboratory AST method was not associated with VRE colonization estimates for livestock (Table 5).

Although poultry meat had the highest prevalence, food subgroup was not associated with VRE colonization prevalence (Tables 4 and 5). Continent of sample origin was the only characteristic associated with VRE colonization prevalence of food products (Table 5). Prevalence of VRE contamination in food products was higher in Africa compared with Asia (*P* = 0.03), Europe (*P* = 0.01) and North America (*P* = 0.001). However, there were only two prevalence estimates of food products from Africa, published in 2022, from fish and poultry products.

### Association of VRE prevalence along the food chain

**Figure 2.**
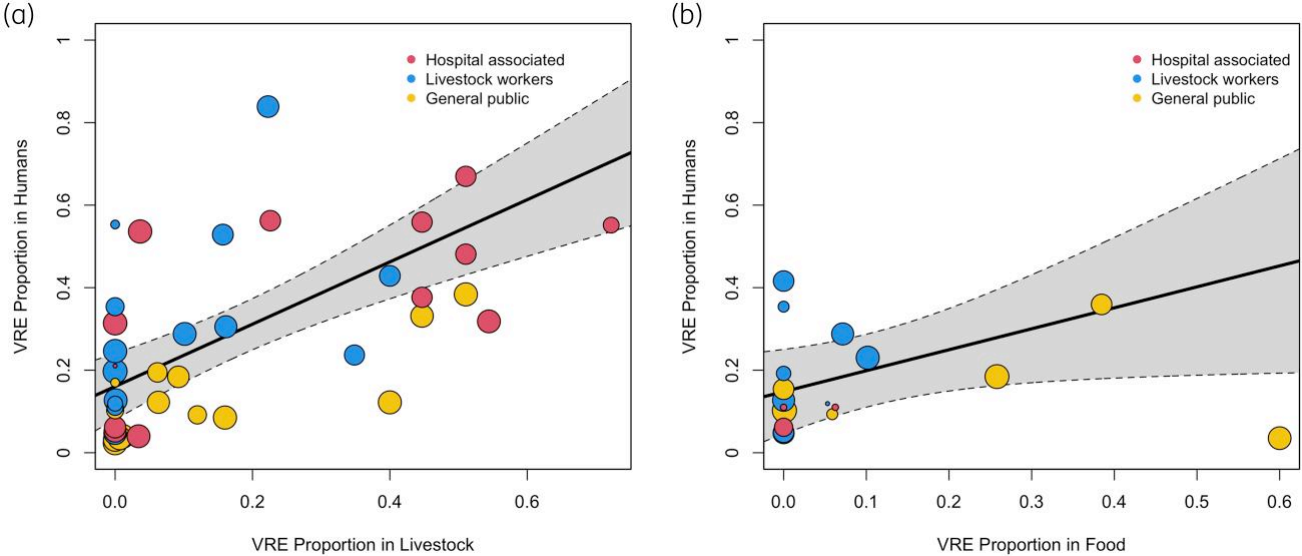
Meta-regression bubble plot of the association of human VRE proportion with (a) VRE proportion in livestock samples and (b) VRE proportion in food samples. Each bubble represents an individual study, size represents study weight in meta-regression analysis, and colour represents human demographic sampled. Solid line represents the regression line surrounded by shaded 95% CI. Livestock workers and slaughterers were combined for clearer visualization.

Human VRE prevalence was associated with VRE in both livestock and in food products (*P* < 0.001, *P* = 0.04, respectively; Table 6). Human VRE prevalence increased at a rate of 0.75% (95% CI: 0.46%–1.04%) per 1% increase in livestock VRE (Table 6 and Figure 2a). The strength of association between human and food product VRE prevalence was lower, with few high prevalence estimates in food. Human VRE prevalence increased at a rate of 0.51% (95% CI: 0.01%–1.00%) per 1% increase in food VRE (Table 6 and Figure 2b).

**Table 6.** Meta-regression of livestock VRE proportion on human VRE (MIC ≥ 32 mg/L) proportion

Variable	Estimate (95% CI)	*P* value
Livestock VRE proportion	0.754 (0.465–1.043)	<0.001
Food VRE proportion	0.508 (0.014–1.002)	0.04

### GRADE evaluation of VRE prevalence estimates

GRADE-based analysis demonstrated that the quality of pooled VRE colonization prevalence estimates varied along the food chain. Human VRE pooled prevalence estimates were of moderate quality, with reduced confidence due to risk of bias, mainly because sample size was justified in only one study, presence of significant heterogeneity and possible publication bias (Table 7). Livestock and food VRE pooled prevalence estimates were both of low quality (Table 7). Livestock estimates had reduced confidence for the same reasons as the human estimates, in addition to limited overlap between CIs of point prevalence estimates. Food VRE colonization pooled prevalence estimates also had imprecision drawbacks, with wide 95% CIs and PIs for each pooled estimate.

**Table 7.** GRADE rating of certainty of evidence for human, food and livestock VRE (MIC ≥ 32 mg/L) prevalence

Outcome	No. of isolates (studies/point estimates)	Quality of evidence (GRADE)	Pooled estimate (95% CI)
Human VRE prevalence	12 678 (36/45)	Moderate^a^ (risk of bias)	0.05 (0.02–0.08)
Food VRE prevalence	4323 (14/17)	Low^b^ (risk of bias, inconsistency and imprecision)	0.06 (0.02–0.11)
Livestock VRE prevalence	4944 (24/39)	Low^c^ (risk of bias, inconsistency)	0.07 (0.01–0.17)

^a^Rare justification of sample size, significant heterogeneity, possible publication bias.

^b^No justification of sample size, significant heterogeneity, publication bias, limited overlap between CIs of point estimates, wide range of pooled estimate CIs and PIs.

^c^Rare justification of sample size, significant heterogeneity, and limited overlap between CIs of point estimates.

## Discussion

In this systematic review and meta-analysis, we estimated the prevalence of VRE in humans, livestock, and food for human consumption. Each group along the food chain had very similar pooled VRE colonization prevalence estimates, ranging from 5% to 7%. This is considerable evidence pointing towards animals and food as potential reservoirs of VRE in the community and their potential to transmit to humans along the food chain. This concept was further supported by a positive association between VRE prevalence in livestock and in humans, both for people with direct contact with animals and the general public. It is likely that the general public are colonized/infected with VRE either through food, contaminated water or some other common exposure shared by livestock and humans. However, clear transmission of human VRE colonization from animals/food cannot be established without consistent observation of identical bacterial strains being isolated from animal and humans, which was not possible with the data available for this systematic review.

As the AMR burden continues increasingly to threaten public health, One Health strategies have been critical in developing action plans to fully understand complex AMR dynamics.^28^ Source attribution of AMR remains difficult to elucidate; however, this systematic review and meta-analysis highlights key concepts of genotyping and comparative exposure assessments.^29^ Human and livestock VRE prevalences were associated with each other, which provides substantial evidence for transmission or common exposure to VRE for humans and animals. Genotypic information in this systematic review loosely supports transmission of VRE between livestock and the general public and farmers, but inference on magnitude and direction of transmission is limited due to minimal availability of typed strains.

Although this meta-analysis cannot specifically attribute sources of VRE acquisition for each human population, the results build upon existing AMR transmission and source attribution literature. Our results strongly align with a previous meta-analysis that determined the absolute risk reduction in humans upon antimicrobial use restriction in livestock to be approximately 24%, with a concurrent 24%–32% reduction in livestock.^4^ This strong association between AMR risk in livestock and humans is highly similar to the results of our meta-analysis. Indeed, Borgen *et al.*^17^ reported that poultry farmers on farms with high VRE prevalence (due to glycopeptide feed additive) had a 13 times higher risk of VRE carriage than farmers on farms with a low VRE burden. In general, attribution of VRE carriage in the community has not been studied extensively, and therefore we cannot examine the role of potential confounding factors like antibiotic regulations and human-to-human spread, among other variables. However, others have reported that nearly 25% of community ESBL-producing *E. coli* carriage was attributed to food consumption/preparation and contact with farm animals, whereas the remaining portion of community acquisition was mostly sourced to human-to-human spread.^30^ Although lower than our estimates of the association between animal and human VRE prevalence, the food chain remains a vastly important AMR source for community acquisition, and continued investment in One Health strategies is necessary to combat increasing AMR burdens.

Genotypic evidence of VRE transmission along the food chain was low due to both the number of reporting studies and limited overlap between STs and PFGE profiles of *Enterococcus* spp. Whereas most typed isolates from this meta-analysis were genetically dissimilar, there are several accounts of shared VRE strains isolated across the food chain.^31–38^ Other reports show a similar trend of host adaptation of VRE strains. Gouliouris *et al.*^39^ performed extensive WGS of vancomycin-resistant *E. faecium* strains isolated across the One Health spectrum in the UK and identified limited overlap in core genomes between livestock faecal and human bloodstream isolates. However, there are some reports of *E. faecium* isolated from animals that cluster within the hospital-associated A1 clade.^32,39,40^ Outside of nosocomial-associated strains, livestock strains are relatively more frequently related to human commensal and wastewater-associated clusters.^8,41,42^ Potentially more worrying, *E. faecalis* appears less host specific than *E. faecium* and displays more extensive strain similarity between livestock and healthy or infected humans.^15,43,44^ WGS analysis of 103 vancomycin-resistant *E. faecalis* ST108 isolates in New Zealand showed fewer than 100 SNPs difference between poultry and human clinical isolates, suggesting recent spread between both hosts.^45^ Despite the low genetic identity between animal, food and nosocomial-adapted *Enterococcus* strains, there is a substantial risk of community acquisition of VRE, even from non-nosocomial strains. Transmission of vancomycin resistance determinants from poultry-associated *E. faecium* ST245 to nosocomial-associated *E. faecium* clonal complex 17 was observed in mice less than 24 h after inoculation.^46^ Conjugative plasmids transferring vancomycin resistance can also simultaneously transmit resistance determinants to other antimicrobial classes, such as macrolide resistance, which has been seen in livestock and human clinical isolates in Taiwan.^38^ Even though strain typing does not fully support direct clinical risk along the food chain, with the possible exception of *E. faecalis*, the relative promiscuity of *Enterococcus* vancomycin resistance determinants warrants stringent protection of our food supply.

Although other meta-analyses have analysed VRE in certain parts of the food chain separately,^18,47^ to our knowledge no other systematic review and meta-analysis has directly compared VRE prevalence across the food chain. Because of the inclusion criteria in our study (studies must have estimated prevalence in humans plus animals or food), we did not capture literature reporting on VRE from only one section of the food chain and this exclusion reduced the power of our meta-analysis. However, our approach enabled a meta-regression to determine the association of VRE along the food chain. This type of analysis is a powerful tool to examine potential AMR transmission dynamics across One Health domains, but still has some biases in its estimates. For example, high heterogeneity in prevalence meta-analyses could have biased these estimates along the food chain, therefore caution must be used when comparing VRE prevalence directly between groups. The intention of using a vancomycin MIC of ≥32 mg/L as one of our inclusion criteria for meta-analysis was to limit possible sources of heterogeneity, but it likely lowered our pooled prevalence estimates by excluding *vanC*-harbouring isolates that exhibit lower-level vancomycin resistance and some VRE with *vanB*-mediated variable vancomycin resistance.^15^ However, these exclusions were made uniformly across human-, food- and animal-derived isolates and were unlikely to substantially affect our estimates of VRE association along the food chain estimates. Additionally, some studies selectively reported *E. faecium* and/or *E. faecalis*; however, these two species are overwhelmingly the most prevalent VRE with an MIC ≥32 mg/L,^15^ so this may not have had a large impact on our prevalence estimates. Otherwise, likely confounding variables such as regional-specific antimicrobial usage and legislative restrictions were not possible to include and could be substantial confounders in our estimate.

Most of the studies included in this systematic review originated from Europe, which was expected as until 1995 avoparcin was used in animal feed additives primarily in poultry and swine operations as a growth promoter.^48^ As such, several European studies were included in our review with relatively large sample sizes and high VRE prevalence. The apparent decrease of European livestock (primarily poultry) VRE prevalence observed in this meta-analysis is well supported by the literature after avoparcin use was discontinued.^19,49,50^ However, some of the most recently published literature included in this review was from African countries, demonstrating fairly high VRE prevalence, suggesting that VRE in the food chain may disproportionally burden lower- and middle-income countries (LMIC).^16,51^ Other systematic reviews echo a high VRE prevalence in some LMIC food chains, but also in companion animals and in environmental water samples.^18,47,52^ Further studies are strongly encouraged to use interdisciplinary approaches to capture VRE data from humans, livestock and other animal species, and environmental samples, ultimately expanding our knowledge of VRE transmission frameworks.

Examining the role of agriculture and food-production animals in human health is a continually evolving area of research that is much needed in today’s AMR landscape. Complex AMR transmission dynamics require serious investment to fully comprehend, and innovative approaches towards source attribution are necessary to develop effective mitigation strategies. This meta-analysis presents a unique approach of meta-regression across study-specific prevalence estimates to determine VRE association and persistence throughout the food chain. This approach could be beneficial towards other high-priority antimicrobial-resistant organisms, such as MRSA and ESBL-producing Enterobacteriaceae. Increased investment in WGS in integrated surveillance protocols across One Health sectors is crucial for accurately determining risks of AMR from the food supply. Risk of AMR acquisition in human populations could be modelled after adjustment for strain-specific genotypes, allowing for development of targeted risk management protocols. Finally, including environmental assessments of VRE with this meta-analysis approach would provide a more complete One Health understanding of resistance dynamics for *Enterococcus* spp., aiding in source attribution of antimicrobial-resistant colonization and infections.

### Conclusions

This meta-analysis identified a continued threat of VRE to global food security and describes the role One Health has in human health and combating AMR. In conclusion, it provides evidence for livestock and food as a reservoir of VRE that has potential to transmit to humans. VRE colonization prevalence was determined to be comparable between humans, livestock and food. Human VRE prevalence was positively associated with increased livestock VRE prevalence, indicating a role for the One Health paradigm in VRE dissemination. Increased WGS and subsequent strain typing is necessary to determine the mechanism and frequency of transmission of VRE and other antimicrobial-resistant pathogens across the food chain.

## Supplementary Material

dkaf008_Supplementary_Data

## Supplementary data

Figures S1 to S6, Tables S1 to S4, and Supplementary material are available as Supplementary data at *JAC* Online.
