# Supplementary material for: Vancomycin-resistant Enterococcus prevalence and its association along the food chain: a systematic review and meta-analysis
Source: J Antimicrob Chemother. 2025 Jan 24;80(4):908–18. doi: 10.1093/jac/dkaf008 (PMC11962378; doi:10.1093/jac/dkaf008)
Supplement: dkaf008_Supplementary_Data [file dkaf008_supplementary_data.docx]

**Supplementary Table S1: Search strategy of MEDLINE database using Ovid**

| # | Search |
| --- | --- |
| 1. | (cattle* or cow* or bovine* or beef or dairy or feedlot* or slaughterhous* or abattoir* or steer* or calf or calves or bos taurus or bos indicus or holstein or heifer or bull or gyr or jersey or brown Swiss or Angus or Piedmontese or Brahman).kf,tw. |
| 2. | exp Cattle/ |
| 3. | exp Dairying/ |
| 4. | cattle diseases/ or mastitis, bovine/ |
| 5. | 1 or 2 or 3 or 4 |
| 6. | exp Vancomycin-Resistant Enterococci/ |
| 7. | exp Vancomycin Resistance/ |
| 8. | exp Enterococcus/ |
| 9. | "enterococc*".kf,tw. |
| 10. | (vancomycin adj2 resistan* adj2 Enterococc*).kf,tw. |
| 11. | ((vancomycin or vancomycin resistan* or glycopeptide) adj2 resistan*).kf,tw. |
| 12. | vre.kf,tw. |
| 13. | 7 or 11 |
| 14. | 6 or 10 or 12 |
| 15. | 8 or 9 |
| 16. | 13 and 15 |
| 17. | 14 or 16 |
| 18. | milk/ or exp dairy products/ |
| 19. | exp Red Meat/ |
| 20. | (milk or cheese* or yogurt or yoghurt or cream or butter).kf,tw. |
| 21. | 18 or 19 or 20 |
| 22. | 5 or 21 |
| 23. | ("vanA" or "vanB" or "vanC" or "vanD" or "vanE").kf,tw. |
| 24. | 17 or 23 |
| 25. | exp Swine/ |
| 26. | exp Poultry/ |
| 27. | exp Ruminants/ |
| 28. | exp Fishes/ |
| 29. | exp Seafood/ or exp Shellfish/ |
| 30. | exp Crustacea/ or exp Aquaculture/ or exp Mollusca/ |
| 31. | (food animal* or farm* or pork* or buffalo* or bison* or sheep* or goat* or lamb* or ewe* or pig*).kf,tw. |
| 32. | exp Poultry Products/ |
| 33. | exp Pork Meat/ |
| 34. | (raw adj2 meat*).kf,tw. |
| 35. | production animal.kf,tw. |
| 36. | 25 or 26 or 27 or 28 or 29 or 30 or 31 or 32 or 33 or 34 or 35 |
| 37. | 22 or 36 |
| 38. | exp Horses/ |
| 39. | exp Livestock/ |
| 40. | exp Sheep/ or exp Sheep, Domestic/ |
| 41. | exp Goats/ |
| 42. | exp Rabbits/ |
| 43. | exp Deer/ |
| 44. | exp food-processing industry/ or exp meat-packing industry/ |
| 45. | (ovine or cervid or game or ruminant or livestock or deadstock or hog* or boar or broiler* or hatcher* or fowl or avian or fish or production animal*).kf,tw. |
| 46. | (salmon or trout or pike or snapper or tuna or shrimp or perch or oyster or tilapia or carp or catfish or halibut or haddock or scallop or mussel or clam).kf,tw. |
| 47. | 37 or 38 or 39 or 40 or 41 or 42 or 43 or 44 or 45 or 46 |
| 48. | 24 and 47 |

**Supplementary Table S2: Study characteristics of included literature**

| **Citation** | **Study aims** | **Study findings** | **Country** | **Sample type^a^** | **Culture target** | **AST method** | **Vancomycin-resistance MIC cut-off^b^** |
| --- | --- | --- | --- | --- | --- | --- | --- |
| Aarestrup et al. 2000 | Investigate enterococci AMR prevalence in the public and farm animals | Similar AMR and resistance gene profiles were found across pig, poultry, and human-derived enterococci | Denmark | H: faecal  A: faecal | All *Enterococcus* & selective VRE enrichment | Broth microdilution & selective VRE agar | 32 |
| Aarestrup et al. 1996 | Determine correlation between avoparcin and vancomycin resistance in livestock | Avoparcin use is positively correlated with VRE colonized farm animals | Denmark | H: faecal  A: faecal | All *Enterococcus* | Agar dilution | 32 |
| Agerso et al. 2008 | Describe VRE prevalence in Danish food products and humans | VRE described in Danish turkey sampling for the first time | Denmark | H: faecal  F: meat | *E. faecalis* & selective VRE enrichment | Broth microdilution & selective VRE agar | 16 |
| Ahmad et al. 2022 | Explore conjugal transfer of VRE to S. aureus | Conjugal transfer inhibited by N. sativa oil | Egypt | H: clinical  A: milk  F: meat | All *Enterococcus* | Disk diffusion | 32 |
| Attia et al. 2017 | Determine prevalence of VRE from different sources | *Enterococcus* are widely distributed with vanA/B genes in chicken samples | Egypt | H: clinical  A: milk  F: meat | All *Enterococcus* | Disk diffusion | 32 |
| Aun et al. 2021 | Characterize *Enterococcus* strains from different hosts | Some *E. faecalis* strains isolated from different hosts are genetically similar, and resistance genes are positively associated with MGEs | Estonia | H: clinical, faecal  A: faecal | All *Enterococcus* & selective VRE enrichment | E-test & selective VRE agar | 32 |
| Van den Bogaard et al. 1997a | Determine if antimicrobial feed additives selects for high carriage of VRE in animals | VRE is present in high levels in humans and animals | The Nether-lands | H: faecal  A: faecal | Selective VRE enrichment | Selective VRE agar | 16 |
| Borgen et al. 2000 | Determine if VRE prevalence remains high in farms that used avoparcin | VRE prevalence is significantly higher in farms and farmers that used avoparcin | Norway | H: faecal  A: faecal | Selective VRE enrichment | Selective VRE agar | 32 |
| Coque et al 1996 | Determine the presence of VRE in the community and the environment | VRE prevalence and subtypes are different between hospital populations and the community/environment | USA | H: faecal  F: meat | Selective VRE enrichment | Selective VRE agar | H: 32  A: 6 |
| De Niederhausern et al. 2007 | To determine VRE reservoirs and investigate potential transfer of vanA genes | vanA type resistance was demonstrated in animals and human samples, and vanA resistance in both types of samples was present on transferable plasmids | Italy, Poland, Hungary, France, Romania, Croatia | H: clinical  A: faecal | All *Enterococcus* & selective VRE enrichment | Selective VRE agar | 32 |
| Donabedian et al. 2010 | To study the distribution of VRE among farm animals and handlers | VRE was identified in swine populations but not among the animal handlers | USA | H: faecal  A: faecal | Selective VRE enrichment | Selective VRE agar | 16 |
| Drahovska et al. 2004 | To compare AMR and virulence factors of enterococci isolate from sheep’s cheese and humans | AMR levels were higher in human clinical isolates compared to cheese isolates. Enterococci from human clinical samples also more often contained hemolysin resistance genes. | Slovakia | H: clinical  F: dairy | All *Enterococcus* | VITEK system | Not stated |
| Eisner et al. 2005 | Study the prevalence of VRE in animals and healthy humans to determine the lingering effect of avoparcin feed | VRE carriage in humans was lower than animals and primarily vanC type compared to vanA for animals | Austria | H: faecal  A: faecal | Selective VRE enrichment | Selective VRE agar | 6 |
| Gambarotto et al. 2001 | Determine the VRE prevalence from retail foods in an area with high human VRE colonization | VRE was detected in high amounts in retail meats, primarily vanC-type similar to the human isolates | France | H: faecal  F: meat | All *Enterococcus* & selective VRE enrichment | Selective VRE agar | 6 |
| Getachew et al. 2012 | Determine the risk factors for VRE colonization in people working with animals in Malaysia | Risk factors associated with VRE carriage for farm workers were age (older has higher risk) and previous hospitalization increased risk | Malaysia | H: faecal  A: faecal | Selective VRE enrichment | Selective VRE agar | 32 |
| Golob et al. 2019 | Compare AMR and virulence gene patterns of *Enterococcus* isolates between human and red meat sources | VRE were not detectable in either human or food specimens, but virulence gene profiles for *E. faecalis* were similar across sample types | Slovenia | H: clinical  F: meat | Selective VRE enrichment | Selective VRE agar | 32 |
| Gonzalez et al. 2009 | To observe AMR patterns and genetic relatedness of enterococci strains isolated from humans, poultry, and the environment | All isolates were susceptible to vancomycin. Ampicillin resistance was common in clonally related enterococci strains isolated from human clinical samples | Spain | H: faecal  A: faecal | *E. faecium* | Selective VRE agar | 6 |
| Hasman et al. 2002 | To describe copper resistance levels and genetic linkage to other resistance phenotypes in *Enterococcus faecium* | tcrB copper resistance gene is genetically linked to van and erm genes from pig sourced *Enterococcus* | Denmark | H: faecal  A: faecal | *E. faecium* | Broth microdilution | 32 |
| Gupta et al. 2007 | To study the prevalence and characteristics of bacteriocin-producing *Enterococcus* from human and dairy product sources | Vancomycin-resistance was present at low rates in bacteriocin-producing *Enterococcus* | India | H: faecal  F: dairy | All *Enterococcus* | Disk diffusion | 32 |
| Borck Hog et al. 2018 | To characterize the AMU and AMR rates of clinically relevant and zoonotic bacteria in Denmark | AMU generally decreased in animals and humans, with associated slight decreases or stagnation in overall AMR among animals and humans | Denmark | H: clinical  A: faecal | All *Enterococcus* | Broth microdilution | 32 |
| Jimenez et al. 2013 | Characterize the enterococci present in milk of different animals compared to humans | Enterococci from milk of animals and humans show genetic diversity across sample sources | Spain | H: milk  A: milk | All *Enterococcus* | E-test | 32 |
| Katakweba et al 2018 | To characterize the AMR profile of enteric indicator bacteria among livestock and humans in Tanzania | Resistance to common antibiotics were abundant in *E. coli* isolates, vancomycin and HLGR were rare in *Enterococcus* | Tanzania | H: faecal  A: faecal | All *Enterococcus* & selective VRE enrichment | Selective VRE agar | 30 |
| Kateete et al. 2013 | Describe the relationship between AMR profiles and genotypes of bacteria from clinical mastitis and milkmen | Similar bacteria and resistance profiles were detected across humans and animals, but bacterial genotypes showed that direct transmission was unlikely | Uganda | H: skin  A: milk | All bacteria | Broth microdilution | 16 |
| Klare et al. 1999 | To describe the effect of discontinuing avoparcin use on farms on human carriage of VRE | Prevalence of VRE carriage in humans was reduced within the following 3 years of avoparcin ban | Germany | H: faecal  F: meat | All *Enterococcus* & selective VRE enrichment | Selective VRE agar | 50 |
| Klare et al. 1995 | To characterize animal products as a reservoir of VRE | VRE were present in meat samples and in human fecal samples, with vanA-type resistance present across sample origins | Germany | H: faecal  F: meat | All *Enterococcus* & selective VRE enrichment | Selective VRE agar | 50 |
| Kolar et al. 2005 | To monitor prevalence of VRE in humans and animals in the Czech Republic | VRE carriage was rare among humans and poultry, and isolates were genotypically distinct across sample sources | Czech Republic | H: faecal  A: faecal | All *Enterococcus* | Broth microdilution | 32 |
| Madoshi et al. 2018 | To characterize *Enterococcus* spp. from human and animal sources | VRE were isolated from cattle and human faecal samples in Tanzania | Tanzania | H: faecal  A: faecal | All *Enterococcus* | Disk diffusion | 30 |
| Mannu et al. 2003 | To compare *Enterococcus faecium* across animal sources and humans | Cheese and sheep enterococcal isolates have lower resistance prevalence and possess fewer virulence determinants than clinical isolates | Italy | H: clinical  A: faecal  F: dairy | *E. faecium* | Broth microdilution | 32 |
| Mellmann et al. 2000 | To investigate livestock as possible sources of VRE infection | VRE was only detectable in hospitalized patients, and thus nosocomial transmission is the most likely cause of VRE infection | Austria | H: faecal  A: faecal | Selective VRE enrichment | Selective VRE agar | 50 |
| Mete et al. 2006 | To characterize the distribution of VRE among livestock and farmers | VRE was not isolated from cattle or their handlers | Turkey | H: faecal  A: faecal | All *Enterococcus* & selective VRE enrichment | Selective VRE agar | 30 |
| Poeta et al. 2005 | To study the prevalence of VRE colonization in the gut of animals and humans | VRE vanA-type were detected in food animals and pets, in addition to identification in healthy humans | Portugal | H: faecal  A: faecal | Selective VRE enrichment | Selective VRE agar | 32 |
| Ribeiro et al. 2007 | To identify reservoirs of VRE and characterize the resistance gene profile of VRE | VRE was not detectable in sheep dairy products, vanA presence did not accurately predict VRE status | Portugal | H: clinical  F: dairy | All *Enterococcus* | E-test | 32 |
| Rice et al. 2003 | To determine the rates of vanC-type vancomycin resistance in enterococci from animals and humans | vanC-type VRE were detectable in all animal species tested, however, high level resistance to vancomycin was not present | USA | H: faecal  A: faecal | Selective VRE enrichment | Selective VRE agar | 4 |
| Richter et al. 2011 | To quantify the occurrence of VRE and MRSA colonization in turkey farms in Germany | Turkey samples appeared to have high percentage of VRE and MRSA, whereas the associated farm worker samples had a high prevalence of MRSA but not VRE | Germany | H: skin  A: faecal | Selective VRE enrichment | Selective VRE agar | Not stated |
| Sabia et al. 2007 | To characterize enterococci isolated from animals, meat, and clinical sources | Enterococci are a genetically diverse group, with many strains across the food chain being vancomycin-resistant | Italy | H: clinical  A: faecal  F: meat | Selective VRE enrichment | Selective VRE agar | 16 |
| Schwaiger et al. 2012 | To describe the presence of vanC1 and pbp5 in *E. faecalis* and characterize associated AMR patterns from pigs and humans | Presence of pbp5 and vanC1 was shown in isolates of pig and human origin | Germany | Not stated | *E. faecalis* | Broth microdilution | 4 |
| Seong et al. 2004 | To determine the extent of gut VRE colonization among humans and chickens, and to characterize the genetic relatedness among VRE isolates from different sources | VRE was present in human and chicken samples, molecular subtyping of VRE suggests that transmission between chickens and humans is possible | Korea | H: faecal  A: faecal | Selective VRE enrichment | Selective VRE agar | 6 |
| Sergelidis et al. 2013 | To characterize the antibiotic resistance patterns in fish markets in northern Greece | Multidrug resistance in enterococci was common across sample sources, however vancomycin-resistance was not detected. High level gentamycin resistance was observed in fish swabs | Greece | H: skin  F: skin | All *Enterococcus* | Broth microdilution | 32 |
| Song et al. 2005 | To determine the potential for VRE transmission across the food chain | VRE was present across the food chain, albeit was only detectable in one healthy human sample compared to a high prevalence from chicken and meats | Korea | H: faecal  A: faecal  F: meat | Selective VRE enrichment | Selective VRE agar | 8 |
| Sorum et al. 2006 | To study the impact of avoparcin ban on VRE prevalence in chicken and farmers | Avoparcin ban was associated with a reduced colonization in both poultry and farmers, sequence typing of VRE confirmed possible transmission across production food chain | Norway | H: faecal  A: faecal | Selective VRE enrichment | Selective VRE agar | 32 |
| Stobberingh et al. 1999 | To assess the potential spread of VRE along the turkey production chain | VRE were isolated from turkeys, turkey-workers, and urban residents. Sequence typing showed heterogenous strain types of VRE but showed some similar strains across the production line. | The Netherlands | H: faecal  A: faecal | Selective VRE enrichment | Selective VRE agar | 32 |
| Telli et al. 2021 | Study the dissemination of VRE and MRSA in beef and chicken slaughterhouses | VRE and MRSA were detected from meat samples, both in the slaughterhouse and in retailers, but were not detectable on slaughterhouse worker hands | Turkey | H: skin  F: meat | All *Enterococcus* | Broth microdilution | 32 |
| Thu et al. 2019 | To describe the prevalence and characteristics of *Enterococcus* in pigs, pork meat, and humans in Thailand and Laos | High-level gentamicin/stretomycin resistance was observed across all samples types, however, no resistance to vancomycin was detectable | Thailand, Laos | H: faecal  F: meat | All *Enterococcus* | Agar dilution | 32 |
| Tzavaras et al. 2012 | To investigate the prevalence of VRE colonization across the poultry production line, and explore genetic relatedness of VRE isolates from animals and humans | VRE was present across the poultry production line, even years after avoparcin ban. PFGE analysis showed that poultry, slaughterhouse workers, and clinical VRE are genetically dissimilar | Greece | H: faecal  A: faecal | Selective VRE enrichment | Selective VRE agar | 32 |
| Van den Bogaard et al. 1997b | To study the relationship between avoparcin use on turkey farms and VRE carriage in turkeys and humans | VRE prevalence was higher on farms using avoparcin, and some VRE from turkeys and their farmers were genetically similar by PFGE | The Netherlands | H: faecal  A: faecal | Selective VRE enrichment | Selective VRE agar | 32 |
| Van den Bogaard et al. 2002 | To study the effect of avoparcin feed on VRE colonization across chicken production systems and in chicken-workers | VRE prevalence in *Enterococcus* spp. was higher in broilers (where avoparcin was used), compared to laying-hens. PFGE profiles for some VRE were similar across chickens and their farmers. | The Netherlands | H: faecal  A: faecal | Selective VRE enrichment | Selective VRE agar | 8 |
| Wambui et al. 2018 | To estimate the prevalence of *Enterococcus* in slaughterhouse lines and their associated AMR profiles | *Enterococcus* were isolated from approximately 25% of samples, no isolates were resistant to either ampicillin or vancomycin | Kenya | H: skin  A: carcass | All bacteria | Disk diffusion | 32 |
| Werner et al. 2000 | To describe the relationship between virginiamycin feed additive and quinipristin/dalfopristin resistance in *Enterococcus* along the food chain | QDRE were isolated from all sources along the food chain, with a similar genotypic profile for resistance | Germany | H: clinical  A: faecal  F: meat | Quinupristin/ dalfopristin resistant *Enterococcus* | Broth microdilution | 16 |
| Wozniak-Biel et al. 2019 | To characterize phenotypic and genotypic AMR traits of *Enterococcus* among humans and turkeys in Poland | AMR was high in both sample origins, with the majority of isolates being resistant to tetracycline and erythromycin | Poland | H: faecal  A: faecal | All *Enterococcus* | Disk diffusion | 32 |
| Youseff et al. 2019 | To characterize the rate of *Enterococcus* colonization among poultry and humans, and describe the AMR phenotype and genotype | Species distributions and AMR profiles of *Enterococcus* were similar across poultry and human sources | Egypt | H: clinical  A: faecal | All *Enterococcus* | Disk diffusion | 32 |
| Zaheer et al. 2020 | To study the potential for beef cattle to transmit AMR bacteria to humans, and determine environmental reservoirs of AMR indicator bacteria | *Enterococcus* species distribution and AMR patterns varied between human and beef isolates | Canada | H: clinical  A: faecal  F: meat | All *Enterococcus* | Disk diffusion | 32 |
| Zhang et al. 2014 | To determine AMR prevalence in *E. faecalis* in human and chicken isolates in China | AMR prevalence in *E. faecalis* from human sources are higher than from chicken sources, but remains high for both sample origins | China | H: clinical  A: faecal | *E. faecalis* | Broth microdilution | 32 |
| Pandova et al. 2024 | Investigate the distribution of virulence factors and antibiotic resistance of Enterococcus from different sources | Count of enterococcal virulence factors and antibiotic resistance genes increased from environmental to food to human sourced isolates | Bulgaria | H: milk  F: dairy | All *Enterococcus* | Disk diffusion | 5 |
| Ngdebe et al. 2023 | To study the distribution of linezolid resistance in animal and human enterococcal isolates | Linezolid resistance phenotype and genotype were observed in similar proportions between humans and animals, except poultry | Nigeria | H: faecal  A: faecal | Selective VRE enrichment | Disk diffusion | 4 |

^a^H: human; A: animal; F; food

^b^MIC presented as µg/mL

**Supplementary Table S3: VRE prevalence and type extracted from included literature**

| **Citation** | **Groups^a^** | **Country** | **Isolate/ Colonization/**  **Herd-level estimates** | **Total tested^b^** | **Total VRE (Number MIC > 32 if known)^b^** | **VRE species** | **VRE genetic types^c^** |
| --- | --- | --- | --- | --- | --- | --- | --- |
| Aarestrup et al. 2000 | H: GP  A: Pig, Poultry | Denmark | Isolate/colonization | GP_i_: 163  GP_c_: 38  Pig_Ai_: 190  Poultry_Ai_: 248 | GP_i_: 0 (0)  GP_c_: 1 (1)  Pig_Ai_: 15 (15)  Poultry_Ai_: 12 (12) | GP_c_: *E. faecalis* (100%)  Pig_Ai_: *E. faecium* (100%)  Poultry_Ai_: *E. faecium* (100%) |  |
| Aarestrup et al. 1996 | H: Hosp  A: Pig, Poultry, Cow | Denmark | Isolate/herd | Hosp_i_: 44  Pig_Ah_: 49  Poultry_Ah_: 51  Cow_Ah_: 32 | Hosp_i_: 2 (2)  Pig_Ah_: 10 (10)  Poultry_Ah_: 38 (38)  Cow_Ah_: 0 (0) | Hosp_i_: *E. faecium* (100%)  Pig_Ah_: *E. faecium* (100%)  Poultry_Ah_: *E. faecium* (95%); *E. faecalis* (5%) |  |
| Agerso et al. 2008 | H: GP  F: Poultry | Denmark | Isolate/colonization | GP_c_: 525  Poultry_Fc_: 3114  Poultry_Fi_: 298 | GP_c_: 5 (5)  Poultry_Fc_: 3 (3)  Poultry_Fi_: 3 (3) | GP_c_: *E. faecium* (60%); *E. faecalis* (40%)  Poultry_Fci_: *E. faecalis* (100%) | H/F: *E. faecalis* ST116 |
| Ahmad et al. 2022 | H: Hosp  A: Cow  F: Fish, Poultry | Egypt | Colonization | Hosp_c_: 17  Cow_Ac_: 18  Poultry_Fc_: 99  Fish_Fc_: 14 | Hosp_c_: 14 (14)  Cow_Ac_: 12 (12)  Poultry_Fc_: 55 (55)  Fish_Fc_: 3 (3) | NT |  |
| Attia et al. 2017 | H: GP  A: Cow  F: Fish, Poultry | Egypt | Isolate | GP_i_: 17  Cow_Ai_: 18  Fish_Fi_: 14  Poultry_Fi_: 99 | GP_i_: 0 (0)  Cow_Ai_: 0 (0)  Fish_Fi_: 0 (0)  Poultry_Fi_: 6 (6) | NT |  |
| Aun et al. 2021 | H: Hosp, LW  A: Cow, Poultry, Pig | Estonia | Isolate | Hosp_i_: 8  LW_i_: 5  Cow_Ai_: 5  Pig_Ai_: 4  Poultry_Ai_: 40 | Hosp_i_: 2 (2)  LW_i_: 0 (0)  Cow_Ai_: 0 (0)  Pig_Ai_: 0 (0)  Poultry_Ai_: 0 (0) | Hosp_i_: *E. faecalis* (100%) | Hosp: *E. faecalis* ST774 |
| Van den Bogaard et al. 1997a | H: GP  A: Pig | The Netherlands | Isolate/colonization | GP_i_: 106  Pig_Ai_: 242  GP_c_: 117  Pig_Ac_: 282 | GP_i_: 14  Pig_Ai_: 96  GP_c_: 14  Pig_Ac_: 96 | NT |  |
| Borgen et al. 2000 | H: LW  A: Poultry | Norway | Colonization/herd | LW_c_: 147  Poultry_Ac_: 147  Poultry_Ah_: 147 | LW_c_: 14 (14)  Poultry_Ac_: 80 (80)  Poultry_Ah_: 80 (80) | NT | Different PFGE profiles between LW and Poultry |
| Coque et al 1996 | H: Hosp, GP  F: Poultry | USA | Colonization | Hosp_c_: 100  GP_c_: 104  Poultry_Fc_: 52 | Hosp_c_: 16 (16)  GP_c_: 3 (2)  Poultry_Fc_: 16 (0) | Hosp_c_: *E. faecalis* (25%); *E. faecium* (75%)  GP_c_: *E. faecalis* (33%); *E. gallinarum* (67%)  Poultry_Fc_: *E. gallinarum* (100%) |  |
| De Niederhausern et al. 2007 | H: Hosp  A: Pig | Italy | Isolate/colonization | Hosp_i_: 93  Hosp_c_: 93  Pig_Ai_: 62  Pig_Ac_: 62 | Hosp_i_: 10 (10)  Hosp_c_: 10 (10)  Pig_Ai_: 8 (8)  Pig_Ac_: 8 (8) | Hosp_ic_: *E. faecalis* (40%); *E. faecium* (60%)  Pig_Aic_: *E. faecium* (75%); *E.* spp. (25%) |  |
| Donabedian et al. 2010 | H: LW  A: Pig, Cow, Sheep, Goat | USA | Colonization | LW_c_: 156  Pig_Ac_: 55  Cow_Ac_: 50  Sheep_Ac_: 57  Goat_Ac_: 14 | LW_c_: 0 (0)  Pig_c_: 6 (6)  Cow_c_: 0 (0)  Sheep_c_: 0 (0)  Goat_c_: 0 (0) | Pig: *E. faecium* (100%) | Pig: *E. faecium* ST5/6/185 |
| Drahovska et al. 2004 | H: Hosp  F: Dairy | Slovakia | Isolate/colonization | Hosp_i_: 109  Hosp_c_: 109  Dairy_Fi_: 117  Dairy_Fc_: 117 | Hosp_i_: 0 (0)  Hosp_c_: 0 (0)  Dairy_Fi_: 0 (0)  Dairy_Fc_: 0 (0) | No VRE |  |
| Eisner et al. 2005 | H: GP  A: Cow, Pig, Poultry | Austria | Colonization | GP_c_: 200  Cow_Ac_: 208  Pig_Ac_: 206  Poultry_Ac_: 205 | GP_c_: 12 (1)  Cow_Ac_: 47 (1)  Pig_Ac_: 49 (0)  Poultry_Ac_: 173 (98) | GP_c_: *E. faecium* (8%); *E. gallinarum* (75%); *E.* spp. (17%)  Cow_Ac_: *E. faecium* (2%); *E. gallinarum* (19%); *E.* spp. (79%)  Pig_Ac_: *E. gallinarum* (55%); *E.* spp. (45%)  Poultry_Ac_: *E. faecium* (50%); *E. gallinarum* (41%); *E.* spp. (9%) |  |
| Gambarotto et al. 2001 | H: Hosp  F: RM, Poultry | France | Colonization | Hosp_c_: 239  RM_Fc_: 50  Poultry_Fc_: 9 | Hosp_c_: 20 (12)  RM_Fc_: 30 (6)  Poultry_Fc_: 9 (0) | Hosp: *E. faecium* (18%); *E. gallinarum* (71%); *E.* spp. (11%)  RM: *E. faecium* (18%); *E. gallinarum* (54%); *E.* spp. (28%)  Poultry: *E. gallinarum* (67%); *E.* spp. (33%) | PFGE profiles between human and pork vanA-*E. faecium* were distinct |
| Getachew et al. 2012 | H: LW  A: Pig, Poultry | Malaysia | Colonization | LW_c_: 298  Pig_Ac_: 54  Poultry_Ac_: 236 | LW_c_: 28 (28)  Pig_Ac_: 0 (0)  Poultry_Ac_: 0 (0) | LW_c_: *E. faecalis* (32%); *E. faecium* (43%); *E. gallinarum* (25%) |  |
| Golob et al. 2019 | H: Hosp  F: RM | Slovenia | Colonization | Hosp_c_: 101  RM_Fc_: 141 | Hosp_c_: 0 (0)  RM_Fc_: 0 (0) | No VRE |  |
| Gonzalez et al. 2009 | H: GP, Hosp  A: Poultry | Spain | Isolate/colonization | Hosp_ic_: 20  GP_ic_: 23  Poultry_Aic_: 5 | Hosp_ic_: 0 (0)  GP_ic_: 0 (0)  Poultry_Aic_: 0 (0) | No VRE |  |
| Hasman et al. 2002 | H: GP  A: Cow, Pig, Poultry, Sheep | Denmark | Isolate/colonization | GP_ic_: 29  Poultry_Aic_: 29  Cow_Aic_: 32  Pig_Aic_: 59  Sheep_Aic_: 22 | GP_ic_: 0 (0)  Poultry_Aic_: 3 (3)  Cow_Aic_: 0 (0)  Pig_Aic_: 14 (14)  Sheep_Aic_: 0 (0) | Poultry_Aic_: *E. faecium* (100%)  Pig_Aic_: *E. faecium* (100%) |  |
| Gupta et al. 2007 | H: GP  F: Dairy | India | Isolate/colonization | GP_ic_: 14  Dairy_Fic_: 121 | GP_ic_: 0 (0)  Dairy_Fic_: 4 (4) | NT |  |
| Borck Hog et al. 2018 | H: Hosp.  A: Pig | Denmark | Isolate/colonization | Hosp_ic_: 1471  Pig_Ai_: 55  Pig_Ac_: 295 | Hosp_ic_: 56 (56)  Pig_Ai_: 0 (0)  Pig_Ac_: 0 (0) | Hosp_ic_: *E. faecium* (100%) |  |
| Jimenez et al. 2013 | H: GP  A: Pig, Sheep | Spain | Isolate/colonization | GP_i_: 7  GP_c_: 8  Pig_Ai_: 16  Pig_Ac_: 9  Sheep_Ai_: 5  Sheep_Ac_: 8 | GP_i_: 0 (0)  GP_c_: 0 (0)  Pig_Ai_: 0 (0)  Pig_Ac_: 0 (0)  Sheep_Ai_: 0 (0)  Sheep_Ac_: 0 (0) | No VRE |  |
| Katakweba et al 2018 | H: GP  A: Pig, Cow, Poultry | Tanzania | Isolate/colonization | GP_i_: 40  GP_c_: 97  Pig_Ai_: 40  Pig_Ac_: 97  Cow_Ai_: 80  Cow_Ac_: 137  Poultry_Ai_: 80  Poultry_Ac_: 194 | GP_i_: 1 (1)  GP_c_: 1 (1)  Pig_Ai_: 3 (3)  Pig_Ac_: 5 (5)  Cow_Ai_: 3 (3)  Cow_Ac_: 7 (7)  Poultry_Ai_: 5 (5)  Poultry_Ac_: 15 (15) | NT |  |
| Kateete et al. 2013 | H: LW  A: Cow | Uganda | Isolate/colonization | LW_i_: 8  LW_c_: 31  Cow_Ai_: 16  Cow_Ac_: 97 | LW_i_: 0  LW_c_: 0  Cow_Ai_: 3  Cow_Ac_: 3 | NT |  |
| Klare et al. 1999 | H: GP  F: Poultry | Germany | Colonization | GP_c_: 400  Poultry_Fc_: 31 | GP_c_: 13 (13)  Poultry_Fc_: 8 (8) | NT |  |
| Klare et al. 1995 | H: GP  F: RM | Germany | Colonization | GP_c_: 100  RM_Fc_: 13 | GP_c_: 12 (12)  RM_Fc_: 5 (5) | GP_c_: *E. faecium* (100%)  RM_Fc_: *E. faecium* (100%) |  |
| Kolar et al. 2005 | H: GP  A: Poultry | Czech Republic | Isolate/colonization | GP_i_: 821  GP_c_: 6023  Poultry_Ai_: 527  Poultry_Ac_: 1050 | GP_i_: 9 (9)  GP_c_: 9 (9)  Poultry_Ai_: 11 (11)  Poultry_Ac_: 11 (11) | GP_ic_: *E. faecalis* (11%); *E. faecium* (22%); *E.* spp. (67%)  Poultry_Aic_: *E. faecalis* (36%); *E. faecium* (64%) | PFGE profiles between human and poultry *E. faecium* and *E. faecalis* were distinct |
| Madoshi et al. 2018 | H: LW  A: Cow | Tanzania | Isolate | LW_i_: 227  Cow_Ai_: 165 | LW_i_: 59 (59)  Cow_Ai_: 6 (6) | NT |  |
| Mannu et al. 2003 | H: Hosp.  A: Sheep  F: Dairy | Italy | Isolate | Hosp_i_: 28  Sheep_Ai_: 26  Dairy_Fi_: 40 | Hosp_i_: 3 (3)  Sheep_Ai_: 0 (0)  Dairy_Fi_: 0 (0) | NT |  |
| Mellmann et al. 2000 | H: GP, Hosp.  A: Pig, Cow, Poultry | Austria | Colonization | Hosp_c_: 226  GP_c_: 443  Pig_Ac_: 171  Cow_Ac_: 105  Poultry_Ac_: 47 | Hosp_c_: 13 (13)  GP_c_: 0 (0)  Pig_Ac_: 0 (0)  Cow_Ac_: 0 (0)  Poultry_Ac_: 0 (0) | Hosp_c_: *E. faecalis* (15%); *E. faecium* (85%) |  |
| Mete et al. 2006 | H: LW  A: Cow | Turkey | Isolate/colonization | LW_i_: 96  LW_c_: 111  Cow_Ai_: 65  Cow_Ac_: 72 | LW_i_: 0 (0)  LW_c_: 0 (0)  Cow_Ai_: 0 (0)  Cow_Ac_: 0 (0) | No VRE |  |
| Poeta et al. 2005 | H: GP  A: Poultry | Portugal | Colonization | GP_c_: 73  Poultry_Ac_: 76 | GP_c_: 2 (2)  Poultry_Ac_: 7 (7) | GP_c_: *E. hirae* (100%)  Poultry_Ac_: *E. faecium* (43%); *E. hirae* (14%); *E.* spp. (43%) |  |
| Ribeiro et al. 2007 | H: Hosp.  F: Dairy | Portugal | Isolate | Hosp_i_: 39  Dairy_Fi_: 59 | Hosp_i_: 1 (1)  Dairy_Fi_: 0 (0) | NT |  |
| Rice et al. 2003 | H: GP  A: Cow, Poultry | USA | Colonization | GP_c_: 17  Poultry_Ac_: 17  Cow_Ac_: 12 | GP_c_: 2  Poultry_Ac_: 16  Cow_Ac_: 12 | NT |  |
| Richter et al. 2011 | H: LW  A: Poultry | Germany | Colonization | LW_c_: 59  Poultry_Ac_: 200 | LW_c_: 0  Poultry_Ac_: 54 | NT |  |
| Sabia et al. 2007 | H: Hosp.  A: Pig  F: RM | Italy | Colonization | Hosp_c_: 154  Pig_Ac_: 79  RM_Fc_: 56 | Hosp_c_: 13 (12)  Pig_Ac_: 8 (8)  RM_Fc_: 5 (4) | Hosp_c_: *E. faecalis* (38%); *E. faecium* (54%); *E.* spp. (8%)  Pig_Ac_: *E. faecium* (50%); *E.* spp. (50%)  RM_Fc_: *E. faecalis* (60%); *E. faecium* (20%); *E.* spp. (20%) |  |
| Schwaiger et al. 2012 | H: GP  A: Pig | Germany | Isolate | GP_i_: 246  Pig_Ai_: 238 | GP_i_: 0 (0)  Pig_Ai_: 13 (0) | Pig_Ai_: *E. faecalis* (100%) |  |
| Seong et al. 2004 | H: GP, Hosp.  A: Poultry | Korea | Colonization | Hosp_c_: 54  GP_c_: 166  Poultry_Ac_: 130 | Hosp_c_: 11 (9)  GP_c_: 13 (2)  Poultry_Ac_: 54 (52) | Hosp_c_: *E. faecium* (82%); *E. gallinarum* (18%)  GP_c_: *E. faecium* (15%); *E. gallinarum* (85%)  Poultry_Ac_: *E. faecalis* (22%); *E. faecium* (74%); *E. gallinarum* (4%) | PFGE and REP-PCR of vanA-*E. faecium* show more genetic similarity between poultry and general public (75–95% similar) than poultry and inpatients (<70% similarity) |
| Sergelidis et al. 2013 | H: SL  F: Fish | Greece | Colonization | SL_c_: 20  Fish_Fc_: 150 | SL_c_: 0 (0)  Fish_Fc_: 0 (0) | No VRE |  |
| Song et al. 2005 | H: GP  A: Cow, Pig, Poultry  F: Poultry | Korea | Colonization | GP_c_: 200  Poultry_Ac_: 114  Cow_Ac_: 11  Pig_Ac_: 25  Poultry_Fc_: 15 | GP_c_: 2 (0)  Poultry_Ac_: 18 (1)  Cow_Ac_: 3 (0)  Pig_Ac_: 1 (0)  Poultry_Fc_: 9 (9) | GP_c_: *E.* spp. (100%)  Poultry_Ac_: *E. faecium* (6%); *E. gallinarum* (83%); *E.* spp. (11%)  Cow_Ac_: *E.* spp. (100%)  Pig_Ac_: *E.* spp. (100%)  Poultry_Fc_: *E. faecium* (100%) |  |
| Sorum et al. 2006 | H: LW  A: Poultry | Norway | Colonization/herd | LW_c_: 19  Poultry_Ah_: 18 | LW_c_: 5 (5)  Poultry_Ah_: 13 (13) | Proportion of species not stated | LW: *E. faecium* ST8/26/242/194/ 195  Poultry: *E. faecium* ST8/146/195/9/ 241 |
| Stobberingh et al. 1999 | H: GP, LW, SL  A: Poultry | The Netherlands | Colonization | GP_c_: 117  LW_c_: 47  SL_c_: 47  Poultry_Ac_: 47 | GP_c_: 12 (12)  LW_c_: 13 (13)  SL_c_: 6 (6)  Poultry_Ac_: 21 (21) | GP_c_: *E. faecium* (93%); *E. hirae* (7%)  LW_c_: *E. faecium* (78%); *E. hirae* (12%)  SL_c_: *E. faecium* (80%); *E. hirae* (20%)  Poultry_Ac_: *E. faecium* (86%); *E. hirae* (14%) | 2 sets of PFGE patterns of *E. faecium* match between turkeys and turkey-farmers |
| Telli et al. 2021 | H: SL  F: RM, Poultry | Turkey | Colonization | SL_c_: 20  RM_Fc_: 40  Poultry_Fc_: 40 | SL_c_: 0 (0)  RM_Fc_: 0 (0)  Poultry_Fc_: 5 (5) | NT |  |
| Thu et al. 2019 | H: Hosp, SL  A: Pig  F: RM | Thailand, Laos | Isolate/colonization | Hosp_c_: 111  SL_c_: 65  Pig_Ai_: 129  Pig_Ac_: 160  RM_Fi_: 312  RM_Fc_: 312 | Hosp_c_: 0 (0)  SL_c_: 0 (0)  Pig_Ai_: 0 (0)  Pig_Ac_: 0 (0)  RM_Fi_: 0 (0)  RM_Fc_: 0 (0) | No VRE |  |
| Tzavaras et al. 2012 | H: SL  A: Poultry | Greece | Colonization | SL_c_: 50  Poultry_Ac_: 500 | SL_c_: 14 (14)  Poultry_Ac_: 113 (113) | SL_c_: *E. feacium* (71%); *E. gallinarum* (29%)  Poultry_Ac_: *E. feacium* (64%); *E. gallinarum* (36%) | PFGE of vanA-*E. faecium* don’t overlap |
| Van den Bogaard et al. 1997b | H: GP, LW, SL  A: Poultry | The Netherlands | Colonization | GP_c_: 188  LW_c_: 47  SL_c_: 48  Poultry_Ac_: 47 | GP_c_: 26 (26)  LW_c_: 18 (18)  SL_c_: 10 (10)  Poultry_Ac_: 24 (24) | NT |  |
| Van den Bogaard et al. 2002 | H: SL, LW  A: Poultry | The Netherlands | Colonization | LW_c_: 76  SL_c_: 46  Poultry_Ac_: 75 | LW_c_: 22  SL_c_: 8  Poultry_Ac_: 43 | LW_c_: *E. faecium* (55%); *E. hirae* (36%); *E.* spp. (9%)  SL_c_: *E. faecalis* (25%); *E. faecium* (50%); *E. hirae* (25%)  Poultry_Ac_: *E. faecalis* (7%); *E. faecium* (60%); *E. hirae* (14%); *E.* spp. (19%) | PFGE of *E. hirae* match for one poultry and farmer pair |
| Wambui et al. 2018 | H: SL  A: Cow | Kenya | Isolate/colonization | SL_i_: 25  SL_c_: 90  Cow_Ai_: 14  Cow_Ac_: 60 | SL_i_: 0 (0)  SL_c_: 0 (0)  Cow_Ai_: 0 (0)  Cow_Ac_: 0 (0) | No VRE |  |
| Werner et al. 2000 | H: GP, Hosp  A: Pig, Poultry  F: RM, Poultry | Germany | Isolate | GP_i_: 30  Hosp_i_: 36  Pig_Ai_: 21  Poultry_Ai_: 17  RM_Fi_: 1  Poultry_Fi_: 20 | GP_i_: 0  Hosp_i_: 36  Pig_Ai_: 1  Poultry_Ai_: 0  RM_Fi_: 0  Poultry_Fi_: 1 | NT |  |
| Wozniak-Biel et al. 2019 | H: Hosp.  A: Poultry | Poland | Isolate/colonization | Hosp_ic_: 56  Poultry_Aic_: 51 | Hosp_ic_: 14 (14)  Poultry_Aic_: 8 (8) | Hosp_ic_: *E. faecalis* (57%); *E. faecium* (7%); *E. gallinarum* (36%)  Poultry_Aic_: *E. faecalis* (75%); *E. faecium* (12%); *E. gallinarum*  (13%) |  |
| Youseff et al. 2019 | H: Hosp.  A: Poultry | Egypt | Isolate | Hosp_i_: 74  Poultry_Ai_: 256 | Hosp_i_: 41 (41)  Poultry_Ai_: 57 (57) | Hosp_ic_: *E. faecalis* (27%); *E. faecium* (51%); *E. hirae* (2%); *E.* spp. (20%)  Poultry_Aic_: *E. faecalis* (25%); *E. faecium* (44%); *E. hirae* (3%); *E.* spp. (28%) |  |
| Zaheer et al. 2020 | H: Hosp.  A: Cow  F: RM | Canada | Isolate | Hosp_i_: 153  Cow_Ai_: 1264  RM_Fi_: 38 | Hosp_i_: 2 (2)  Cow_Ai_: 1 (1)  RM_Fi_: 0 (0) | Hosp_i_: *E. faecalis* (100%)  Cow_Ai_: *E. hirae* (100%) |  |
| Zhang et al. 2014 | H: Hosp.  A: Poultry | China | Isolate | Hosp_i_: 30  Poultry_Ai_: 30 | Hosp_i_: 24 (24)  Poultry_Ai_: 11 (11) | Hosp_i_: *E. faecalis* (100%)  Poultry_Ai_: *E. faecalis* (100%) |  |
| Pandova et al. 2024 | H: GP  F: Dairy | Bulgaria | Isolate | GP_i_: 16  F_i_: 38 | GP_i_: 0  F_i_: 0 | No VRE |  |
| Ngdebe et al. 2023 | H: Hosp.  A: Cattle, Pig, Poultry | Nigeria | Colonization | Hosp_c_: 180  Cattle_Ac_: 85  Pig_Ac_: 125  Poultry_Ac_: 150 | Hosp_c_: 0  Cattle_Ac_: 0  Pig_Ac_: 0  Poultry_Ac_: 0 | No VRE |  |

^a^H: human; GP, general public; LW, livestock workers; Hosp, hospital-associated sample; SL, slaughterhouse worker/butcher/retailer; A: animal; F; food; RM, red meat

^b^subscript symbols: A: animal; F: food; i: isolate-level counts; c: colonization-level counts; h: herd-level counts

^c^VRE genetic typing only described if multilocus sequence typing, or if PFGE genetic profile studied for both animal/food and human VRE isolate

**Supplementary Table S4: Quality assessment of included literature**

| **Citation** | **Representativeness** | **Sample Size** | **Outcome Assessment** |
| --- | --- | --- | --- |
| Aarestrup et al. 2000 | 1 | 0 | 2 |
| Aarestrup et al. 1996 | 1 | 0 | 2 |
| Agerso et al. 2008 | 1 | 0 | 0 |
| Ahmad et al. 2022 | 0 | 0 | 2 |
| Attia et al. 2017 | 0 | 0 | 2 |
| Aun et al. 2021 | 1 | 0 | 2 |
| Van den Bogaard et al. 1997 (1) | 0 | 0 | 1 |
| Borgen et al. 2000 | 1 | 0 | 2 |
| Coque et al 1996 | 1 | 0 | 2 |
| De Niederhausern et al. 2007 | 0 | 0 | 2 |
| Donabedian et al. 2010 | 1 | 0 | 1 |
| Drahovska et al. 2004 | 1 | 0 | 2 |
| Eisner et al. 2005 | 1 | 0 | 2 |
| Gambarotto et al. 2001 | 0 | 0 | 1 |
| Getachew et al. 2012 | 1 | 0 | 2 |
| Golob et al. 2019 | 0 | 0 | 2 |
| Gonzalez et al. 2009 | 0 | 0 | 2 |
| Hasman et al. 2002 | 0 | 0 | 1 |
| Gupta et al. 2007 | 0 | 0 | 2 |
| Borck Hog et al. 2018 | 1 | 1 | 2 |
| Jimenez et al. 2013 | 0 | 0 | 2 |
| Katakweba et al 2018 | 1 | 0 | 2 |
| Kateete et al. 2013 | 1 | 0 | 1 |
| Klare et al. 1999 | 1 | 0 | 1 |
| Klare et al. 1995 | 1 | 0 | 1 |
| Kolar et al. 2005 | 1 | 0 | 2 |
| Madoshi et al. 2018 | 1 | 0 | 2 |
| Mannu et al. 2003 | 1 | 0 | 2 |
| Mellmann et al. 2000 | 1 | 0 | 2 |
| Mete et al. 2006 | 1 | 0 | 2 |
| Poeta et al. 2005 | 1 | 0 | 2 |
| Ribeiro et al. 2007 | 1 | 0 | 2 |
| Rice et al. 2003 | 0 | 0 | 1 |
| Richter et al. 2011 | 1 | 0 | 1 |
| Sabia et al. 2007 | 0 | 0 | 2 |
| Schwaiger et al. 2012 | 0 | 0 | 1 |
| Seong et al. 2004 | 1 | 0 | 2 |
| Sergelidis et al. 2013 | 1 | 0 | 2 |
| Song et al. 2005 | 0 | 0 | 2 |
| Sorum et al. 2006 | 1 | 0 | 1 |
| Stobberingh et al. 1999 | 1 | 0 | 2 |
| Telli et al. 2021 | 1 | 0 | 2 |
| Thu et al. 2019 | 1 | 0 | 2 |
| Tzavaras et al. 2012 | 1 | 0 | 2 |
| Van den Bogaard et al. 1997 (2) | 0 | 0 | 1 |
| Van den Bogaard et al. 2002 | 1 | 0 | 2 |
| Wambui et al. 2018 | 1 | 0 | 2 |
| Werner et al. 2000 | 0 | 0 | 1 |
| Wozniak-Biel et al. 2019 | 0 | 0 | 2 |
| Youseff et al. 2019 | 1 | 0 | 2 |
| Zaheer et al. 2020 | 1 | 0 | 2 |
| Zhang et al. 2014 | 1 | 0 | 1 |
| Pandova et al. 2024 | 1 | 0 | 2 |
| Ngdebe et al. 2023 | 1 | 0 | 2 |

Green represent full score; yellow represent a half score; red represents a score of zero.


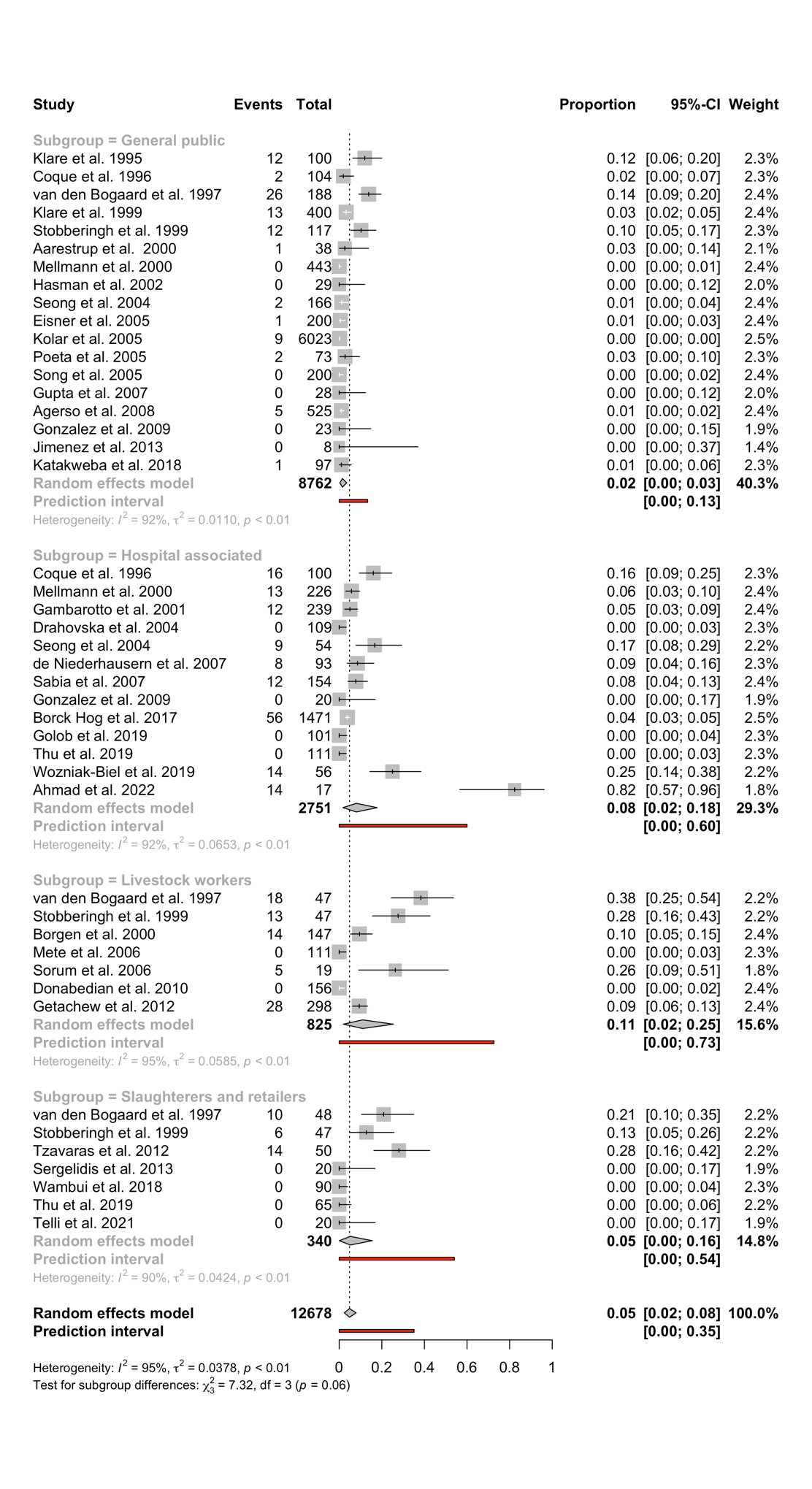


**Supplementary Figure S1: Human VRE pooled prevalence estimates.** Subgroup analysis is based on human population type.


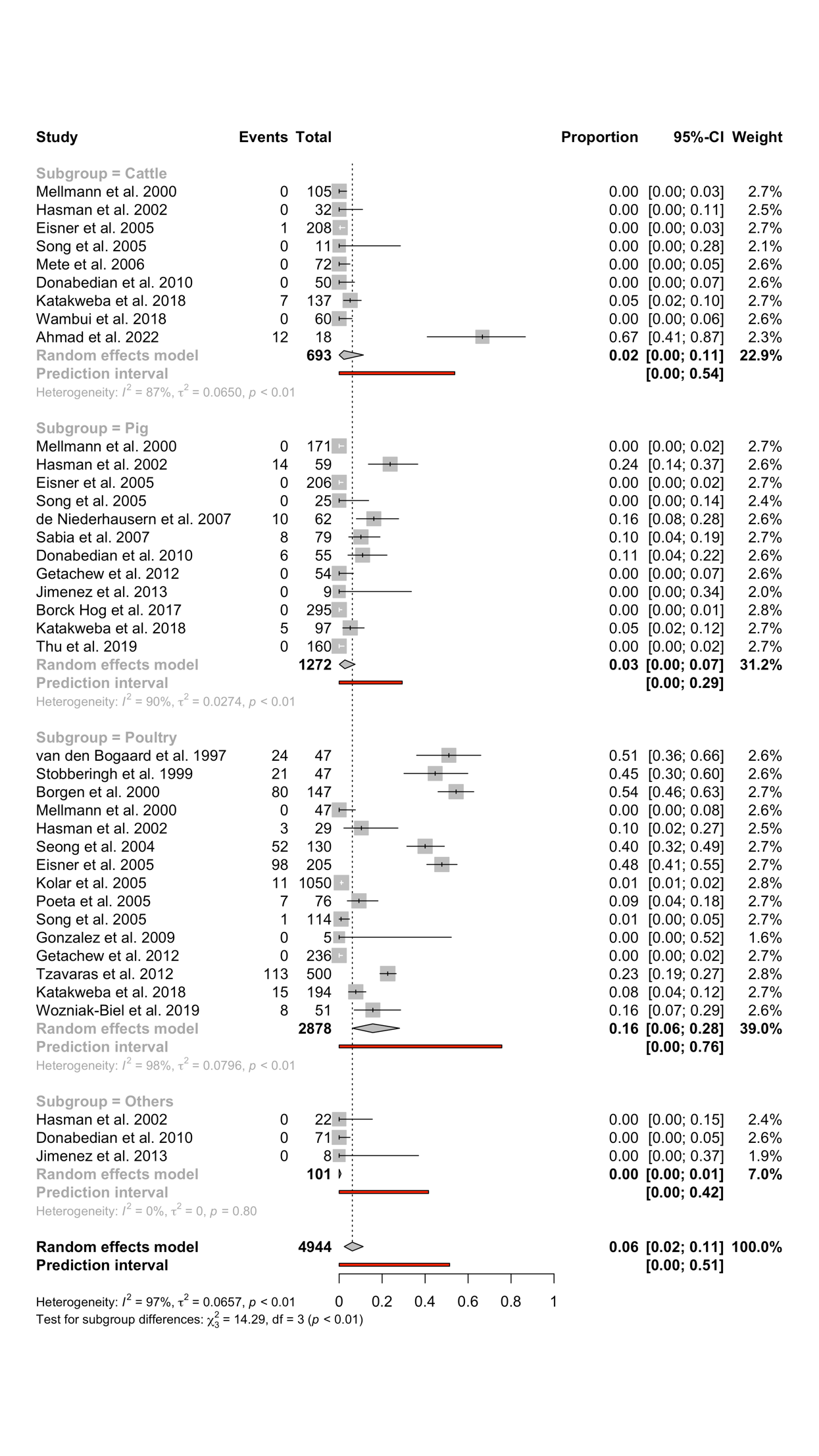


**Supplementary Figure S2: Livestock VRE pooled prevalence estimates.** Subgroup analysis is based on livestock species. Others includes sheep and goat specimens.


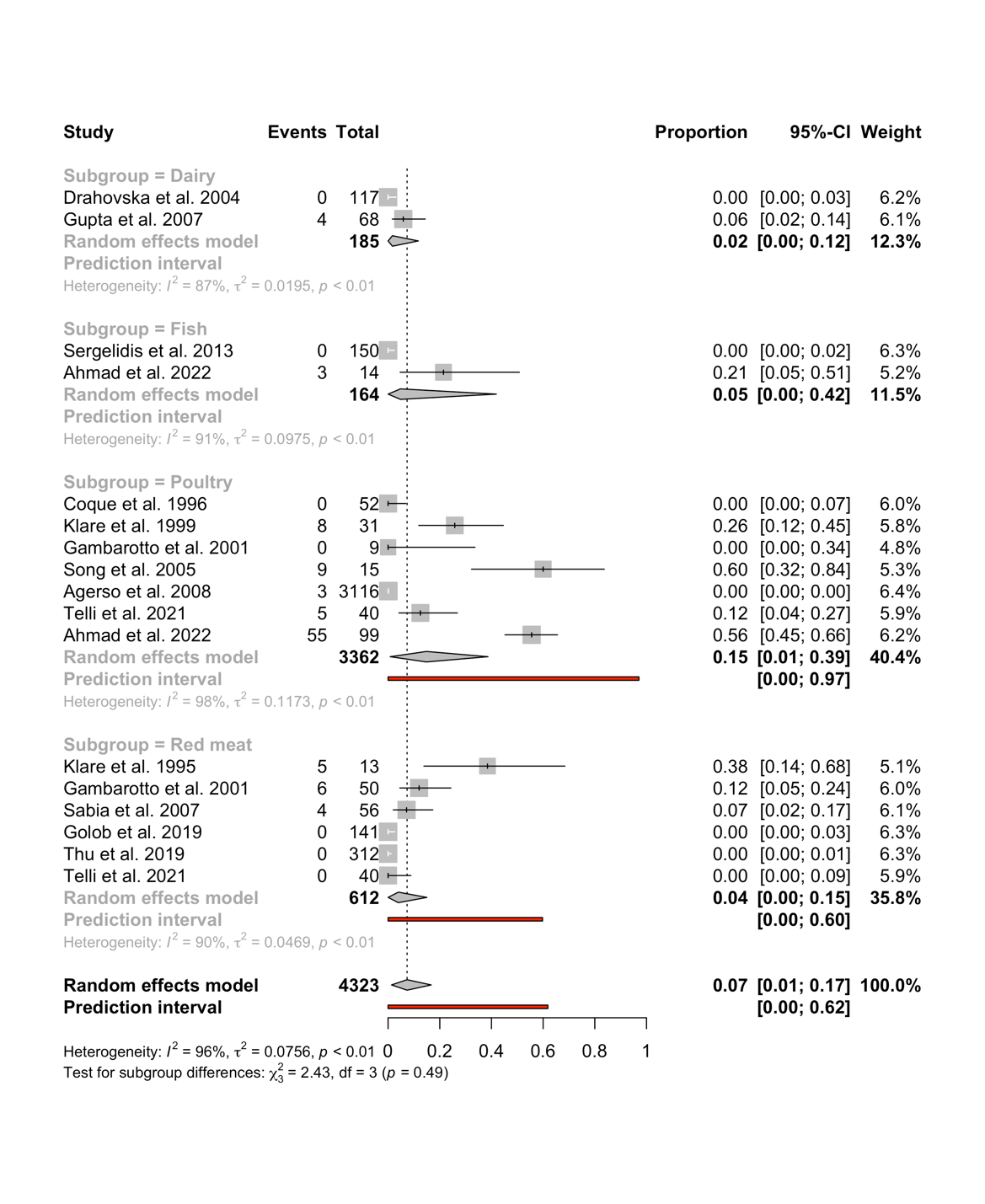


**Supplementary Figure S3: Food VRE pooled prevalence estimates.** Subgroup analysis is based on livestock species.

**Supplementary Figure S4: Funnel plot test for publication bias of human VRE prevalence estimates.** Egger’s test showed significant evidence for publication bias (P < 0.0001).

**Supplementary Figure S5: Funnel plot test for publication bias of livestock VRE prevalence estimates.** Egger’s test showed no significant evidence for publication bias (P = 0.3910).

**Supplementary Figure S6: Funnel plot test for publication bias of food VRE prevalence estimates.** Egger’s test showed significant evidence for publication bias (P < 0.0410).

**Included References:**

Aarestrup FM, Agerso Y, Gerner-Smidt P, Madsen M, Jensen LB. Comparison of antimicrobial resistance phenotypes and resistance genes in *Enterococcus faecalis* and *Enterococcus faecium* from humans in the community, broilers, and pigs in Denmark. Diagn Microbiol Infect Dis 2000; 37: 127–37.

Aarestrup FM, Ahrens P, Madsen M, Pallesen L V, Lykke Poulsen R, Westh H. Glycopeptide susceptibility among Danish *Enterococcus faecium* and *Enterococcus faecalis* isolates of animal and human origin and PCR identification of genes within the vanA cluster. Antimicrob Agents Chemother 1996; 40: 1938–40.

Agersø Y, Lester CH, Porsbo LJ, et al. Vancomycin-resistant *Enterococcus faecalis* isolates from a Danish patient and two healthy human volunteers are possibly related to isolates from imported turkey meat. Journal of Antimicrobial Chemotherapy 2008; 62: 844–5.

Ahmad AAM, Gharib AA, Elshorbgy I, Elewasy OA, Elmowalid GA. Nigella sativa oil extract: A natural novel specific conjugal transfer inhibitor of vancomycin resistance from vanA/B-resistant *Enterococcus faecium* to *Staphylococcus aureus.* J Appl Microbiol 2022; 133: 619–29.

Attia AM, Gharib AA, Mohamed II, Ahmed OE. Phenotypic and genotypic identification of vancomycin resistant enterococci from different sources. Zagazig Vet J 2017; 45: 64–73.

Aun E, Kisand V, Laht M, et al. Molecular characterization of Enterococcus isolates From different sources in Estonia reveals potential transmission of resistance genes among different reservoirs. Front Microbiol 2021; 12: 601490.

van den Bogaard AE, Mertens P, London NH, Stobberingh EE. High prevalence of colonization with vancomycin- and pristinamycin-resistant enterococci in healthy humans and pigs in The Netherlands: is the addition of antibiotics to animal feeds to blame? Journal of Antimicrobial Chemotherapy 1997; 40: 454–6.

Borgen K, Simonsen GS, Sundsfjord A, Wasteson Y, Olsvik O, Kruse H. Continuing high prevalence of VanA-type vancomycin-resistant enterococci on Norwegian poultry farms three years after avoparcin was banned. J Appl Microbiol 2000; 89: 478–85.

De Niederhäusern S, Sabia C, Messi P, Guerrieri E, Manicardi G, Bondi M. VanA-type vancomycin-resistant enterococci in equine and swine rectal swabs and in human clinical samples. Curr Microbiol 2007; 55: 240–6.

Donabedian SM, Perri MB, Abdujamilova N, et al. Characterization of vancomycin-resistant *Enterococcus faecium* isolated from swine in three michigan counties. J Clin Microbiol 2010; 48: 4156–60.

Drahovská H, Slobodníková L, Kocíncová D, et al. Antibiotic resistance and virulence factors among clinical and food enterococci isolated in Slovakia. Folia Microbiol 2004; 49: 763–8.

Eisner A, Feierl G, Gorkiewicz G, et al. High prevalence of vanA-type vancomycin-resistant enterococci in austrian poultry. Appl Environ Microbiol 2005; 71: 6407–9.

Gambarotto K, Ploy MC, Dupron F, Giangiobbe M, Denis F. Occurrence of vancomycin-resistant enterococci in pork and poultry products from a cattle-rearing area of France. J Clin Microbiol 2001; 39: 2354–5.

Getachew Y, Hassan L, Zakaria Z, et al. Characterization and risk factors of vancomycin-resistant enterococci (VRE) among animal-affiliated workers in Malaysia. J Appl Microbiol 2012; 113: 1184–95.

Coque TM, Tomayko JF, Ricke SC, Okhyusen PC, Murray BE. Vancomycin-resistant enterococci from nosocomial, community, and animal sources in the United States. Antimicrob Agents Chemother 1996; 40: 2605–9.

Golob M, Pate M, Kušar D, et al. Antimicrobial resistance and virulence genes in *Enterococcus* *faecium* and *Enterococcus faecalis* from humans and retail red meat. Biomed Res Int 2019; 2019: 1–12.

Gonzalez M, Afonso O, Tejedor MT. Antimicrobial susceptibility and molecular typing of *Enterococcus faecium* isolated from humans, chickens and environment in Canary Islands (Spain). Revista Española de Quimioterapia 2009; 22: 120–6.

Hasman H, Aarestrup FM. tcrb, a gene conferring transferable copper resistance in *Enterococcus faecium*: Occurrence, transferability, and linkage to macrolide and glycopeptide resistance. Antimicrob Agents Chemother 2002; 46: 1410–6.

Gupta H, Malik RK. Incidence of virulence in bacteriocin-producing enterococcal isolates. Lait 2007; 87: 587–601.

Borck Høg B, Bager F, korsgaard HB, et al. DANMAP 2017 - Use of antimicrobial agents and occurrence of antimicrobial resistance in bacteria from food animals, food and humans in Denmark. 2018. Available at: www.danmap.org.

Jiménez E, Ladero V, Chico I, et al. Antibiotic resistance, virulence determinants and production of biogenic amines among enterococci from ovine, feline, canine, porcine and human milk. BMC Microbiol 2013; 13: 288.

Katakweba AAS, Muhairwa AP, Lupindu AM, et al. First report on a randomized investigation of antimicrobial resistance in fecal indicator bacteria from livestock, poultry, and humans in Tanzania. Microbial Drug Resistance 2018; 24: 260–8.

Kateete DP, Kabugo U, Baluku H, et al. Prevalence and antimicrobial susceptibility patterns of bacteria from milkmen and cows with clinical mastitis in and around Kampala, Uganda. PLoS One 2013; 8: e63413.

Klare I, Badstübner D, Konstabel C, Böhme G, Claus H, Witte W. Decreased incidence of vanA-type vancomycin-resistant Enterococci isolated from poultry meat and from fecal samples of humans in the community after discontinuation of avoparcin usage in animal husbandry. Microbial Drug Resistance 1999; 5: 45–52.

Klare I, Heier H, Claus H, et al. *Enterococcus faecium* strains with vanA-mediated high-level glycopeptide resistance isolated from animal foodstuffs and fecal samples of humans in the community. Microbial Drug Resistance 1995; 1: 265–72.

Kolar M, Pantucek R, Bardon J, et al. Occurrence of vancomycin-resistant enterococci in humans and animals in the Czech Republic between 2002 and 2004. J Med Microbiol 2005; 54: 965–7.

Madoshi BP, Mtambo MMA, Muhairwa AP, Lupindu AM, Olsen JE. Isolation of vancomycin-resistant *Enterococcus* from apparently healthy human animal attendants, cattle and cattle wastes in Tanzania. J Appl Microbiol 2018; 124: 1303–10.

Mannu L, Paba A, Daga E, et al. Comparison of the incidence of virulence determinants and antibiotic resistance between *Enterococcus faecium* strains of dairy, animal and clinical origin. Int J Food Microbiol 2003; 88: 291–304.

Mellmann A, Orth D, Dierich MP, Allerberger F, Klare I, Witte W. Nosocomial cross transmission as a primary cause of vancomycin-resistant enterococci in Austria. Journal of Hospital Infection 2000; 44: 281–7.

Mete E, Kaleli I. Species distribution and antibiotic resistance of enterococci isolated from cattle farmers and cattles. Mikrobiyol Bult 2006; 40: 75–80.

Poeta P, Costa D, Rodrigues J, Torres C. Study of faecal colonization by vanA-containing *Enterococcus* strains in healthy humans, pets, poultry and wild animals in Portugal. Journal of Antimicrobial Chemotherapy 2005; 55: 278–80.

Ribeiro T, Abrantes M, Lopes M de FS, Crespo MTB. Vancomycin-susceptible dairy and clinical enterococcal isolates carry vanA and vanB genes. Int J Food Microbiol 2007; 113: 289–95.

Rice EW, Boczek LA, Johnson CH, Messer JW. Detection of intrinsic vancomycin resistant enterococci in animal and human feces. Diagn Microbiol Infect Dis 2003; 46: 155–8.

Richter A. Untersuchungen zum Vorkommen von Methicillin- resistenten Staphylokokken (MRS) und Vancomycin- resistenten Enterokokken (VRE) in Putenmastbetrieben. 2011.

Sabia C, De Niederhäusern S, Guerrieri E, et al. Detection of bacteriocin production and virulence traits in vancomycin-resistant enterococci of different sources. J Appl Microbiol 2008; 104: 970–9.

Schwaiger K, Bauer J, Hörmansdorfer S, et al. Presence of the resistance genes vanC1 and pbp5 in phenotypically vancomycin and ampicillin susceptible *Enterococcus faecalis*. Microbial Drug Resistance 2012; 18: 434–9.

Seong C, Sook Shim E, Moo Kim S, Cheol Yoo J. Prevalence and characterization of vancomycin-resistant enterococci in chicken intestines and humans of Korea. Arch Pharm Res 2004; 27: 246–53.

Sergelidis D, Abrahim A, Papadopoulos T, et al. Antimicrobial susceptibility of *Enterococcus* spp. isolated from freshwater fish and personnel and equipment of fish markets in northern Greece. Journal of the Hellenic Veterinary Medical Society 2013; 64: 239–48.

Kim W-J, Song JY, Hwang IS, et al. Prevalence and molecular epidemiology of vancomycin-resistant enterococci (VRE) strains isolated from animals and humans in Korea. Korean J Intern Med 2005; 20: 55–62.

Sørum M, Johnsen PJ, Aasnes B, et al. Prevalence, persistence, and molecular characterization of glycopeptide-resistant enterococci in Norwegian poultry and poultry farmers 3 to 8 years after the ban on avoparcin. Appl Environ Microbiol 2006; 72: 516–21.

Stobberingh E, Van Den Bogaard A, London N, Driessen C, Top J, Willems R. Enterococci with glycopeptide resistance in turkeys, turkey farmers, turkey slaughterers, and (sub)urban residents in the South of The Netherlands: evidence for transmission of vancomycin resistance from animals to humans? Antimicrob Agents Chemother 1999; 43: 2215–21.

Telli N, Telli AE, Biçer Y, Turkal G, Uçar G. Isolation and antimicrobial resistance of vancomycin resistant *Enterococcus* spp. (VRE) and methicillin-resistant *S. aureus* (MRSA) on beef and chicken meat, and workers hands from slaughterhouses and retail shops in Turkey. Journal of the Hellenic Veterinary Medical Society 2021; 72: 3345–54.

Thu WP, Sinwat N, Bitrus AA, Angkittitrakul S, Prathan R, Chuanchuen R. Prevalence, antimicrobial resistance, virulence gene, and class 1 integrons of *Enterococcus faecium* and *Enterococcus faecalis* from pigs, pork and humans in Thai-Laos border provinces. J Glob Antimicrob Resist 2019; 18: 130–8.

Tzavaras I, Siarkou VI, Zdragas A, et al. Diversity of vanA-type vancomycin-resistant *Enterococcus faecium* isolated from broilers, poultry slaughterers and hospitalized humans in Greece. Journal of Antimicrobial Chemotherapy 2012; 67: 1811–8.

van den Bogaard AE, Jensen LB, Stobberingh EE. Vancomycin-resistant enterococci in turkeys and farmers. New England Journal of Medicine 1997; 337: 1558–9.

van den Bogaard AE, Willems R, London N, Top J, Stobberingh EE. Antibiotic resistance of faecal enterococci in poultry, poultry farmers and poultry slaughterers. Journal of Antimicrobial Chemotherapy 2002; 49: 497–505.

Wambui J, Tasara T, Kamau Njage PM, Stephan R. Species distribution and antimicrobial profiles of *Enterococcus* spp. isolates from Kenyan small and medium enterprise slaughterhouses. J Food Prot 2018; 81: 1445–9.

Werner G, Klare I, Heier H, et al. Quinupristin/dalfopristin-resistant enterococci of the satA {vatD) and satG (vatE) genotypes from different ecological origins in Germany. Microbial Drug Resistance 2000; 6: 37–47.

Woźniak-Biel A, Bugla-Płoskońska G, Burdzy J, Korzekwa K, Ploch S, Wieliczko A. Antimicrobial resistance and biofilm formation in *Enterococcus* spp. Isolated from humans and turkeys in Poland. Microbial Drug Resistance 2019; 25: 277–86.

Youssef A, Ibrahim H, Hamed D, Fawzy D. Human health risks associated with antimicrobial-resistant enterococci isolated from poultry and human. Alex J Vet Sci 2019; 62: 116–29.

Zaheer R, Cook SR, Barbieri R, et al. Surveillance of *Enterococcus* spp. reveals distinct species and antimicrobial resistance diversity across a One-Health continuum. Sci Rep 2020; 10: 3937.

Zhang H, Ye D, He Q, Wang M, Mao T, Shao S. Study of drug resistance and gene distribution of virulent factors in *Enterococcus faecalis* from human and chicken. Modern Preventive Medicine 2014; 41: 2611–6.

Pandova M, Kizheva Y, Tsenova M, Rusinova M, Borisova T, Hristova P. Pathogenic potential and antibiotic susceptibility: A comprehensive study of enterococci from different ecological settings. Pathogens 2024; 13: 36.

Ngbede EO, Sy I, Akwuobu CA, et al. Carriage of linezolid-resistant enterococci (LRE) among humans and animals in Nigeria: coexistence of the cfr, optrA, and poxtA genes in *Enterococcus* *faecium* of animal origin. J Glob Antimicrob Resist 2023; 34: 234–9.
